# Responses of fisheries ecosystems to marine heatwaves and other extreme events

**DOI:** 10.1371/journal.pone.0315224

**Published:** 2024-12-06

**Authors:** Anthony R. Marshak, Jason S. Link

**Affiliations:** 1 National Centers for Coastal Ocean Science, National Ocean Service, National Oceanic and Atmospheric Administration, Silver Spring, Maryland, United States of America; 2 Office of the Assistant Administrator, National Marine Fisheries Service, National Oceanic and Atmospheric Administration, Woods Hole, Massachusetts, United States of America; Aristotle University of Thessaloniki, GREECE

## Abstract

Marine ecosystems and their living marine resources (LMRs) continue to respond to the effects of global change, with environmental factors impacting marine fisheries biomass, distribution, harvest, and associated economic performance. Extreme events such as high-category hurricanes, harmful algal blooms, marine heatwaves, and large-scale hypoxia affect major regions and subregions of United States waters, with their frequency expected to increase over the next decades. The impacts of extreme events on fisheries biomass, harvest, and economic performance have not been examined as closely as a system (i.e., cumulatively), or in terms of their differential effects on particular functional groups of a given system. Among several U.S. subregions, we examined responses of fisheries biomass, landings, and revenue for particular functional groups to large-scale environmental perturbations (i.e., marine heatwaves, Hurricane Katrina, Deepwater Horizon oil spill). Distinct negative short-term consequences to annual fisheries biomass, landings, and revenue were observed in all regions, including at the system-level scale for several ecosystems which have higher proportions of pelagic species composition and variable shellfish-based revenue. In addition, shifts in species composition often were associated with environmental perturbations. Recovery to pre-perturbation levels (both in the immediate years following the event and over the post-event period of study) and resilience at the system level was observed in several cases, although post-event declines in biomass and landings occurred in the California ecosystem. Certain extreme events are expected to become more common in marine environments, with resulting perturbations throughout multiple components of U.S. socioecological systems. The recognition and understanding of the consequences of extreme events throughout marine ecosystems is necessary for effective, holistic, and sustainable management practices.

## Introduction

Marine ecosystems and their living marine resources (LMRs) continue to respond to the effects of global change, with environmental factors affecting these coupled social-ecological systems [1, 2]. Extreme events such as high category hurricanes, harmful algal blooms, marine heatwaves, large-scale hypoxia, and anthropogenic events such as oil and chemical spills affect marine ecosystems [3–7]. The frequency and intensity of such extreme events is expected to increase over the coming decades [6, 8–10]. Yet climate change research tends to explore persistent and chronic effects on ocean ecosystems, or focus on increases in average temperature, but not necessarily significant, acute, and intense perturbations like these extreme events [11–14]. Ecological research tends to explore pulse-press or acute effects on only select components of ecosystems in experimental contexts, but rarely on entire ecosystems across multiple taxa and trophic levels and only rarely beyond experimental scales [15–17]. We need both to truly understand the short- and long-term response and resilience of marine ecosystems to these extreme events [16, 17]. Understanding whether ecosystems exhibit hysteresis or shift to alternate states is crucial as both long-term and extreme event phenomena continue to increase in their impacts on marine ecosystems [18–20]. Many examples document regime shifts in components of marine ecosystems, but do not necessarily examine them over multi-decadal timeframes, nor do they comprehensively cover the key facets of the entire socio-ecological system [15–17, 21, 22].

Typically, assessments of impacts from extreme events to marine ecosystems and their LMRs focus on select components of an ecosystem, particular species or stocks, or for a limited subset of processes being perturbed [16, 21–23]. Although helpful, those more reductionist approaches do not include the larger responses of the suite of LMR taxa as an *entire* system [13–16, 21–23], nor do they examine emergent properties of marine ecosystems that are increasingly insightful [24, 25]. It seems prudent to adopt a wider, multi-tiered scope as extreme events continue to impact marine ecosystems and their LMRs. Because the resources to examine the myriad populations and processes are limited, some form of an integrated or aggregated view will provide a more efficient means to track any changes [22, 26, 27]. Such views hold promise in terms of insights from emergence, earlier detection of responses, and dynamics that impact components of an ecosystem. Such an approach builds on hierarchy theory for complex, adaptive systems and provides an opportunity to see major, systematic impacts much more rapidly than *post-hoc* piecing them together from component taxa and processes [26–30]. As one would predict that higher levels of the biological hierarchy (i.e., at the multispecies, aggregate group, and system levels) are more stable than lower levels, tracking a range of emergent ecosystem features should be able to provide insights that would be missed at lower levels, and also should delineate severity of a perturbation if one were detected at higher levels [31].

Extreme events impact the biomass, production, and harvest of LMRs, as well as the economic performance of their associated fisheries [32–34]. Previous work has examined the foundational limits that primary production sets on fisheries ecosystems, including on total landings, maximum potential revenue, and LMR-based employment [35–38]. However, the impacts of extreme events on biomass, harvest, and revenue have not been evaluated as a fully coupled economic-ecological system or across multiple socioecological systems. Truly ascertaining the impact of extreme events necessitates an examination of any associated or resulting economic impacts to the marine ecosystem.

How extreme events impact LMR dynamics, and their associated economics *in toto* is not always clear, nor are the response times, duration, or any potential recovery from these events well known. Here we examine responses in fisheries biomass (i.e., biomass of fishery and ecosystem component species), landings, and revenue to extreme events over time. We do so for several eastern Pacific (eastern Bering Sea, Gulf of Alaska, California, Pacific NW) and western Atlantic (Gulf of Maine, Gulf of Mexico) ecosystems relative to marine heatwaves and regional warming, a high-category hurricane, and a major oil spill. We examined these responses at the ecosystem level (i.e., total system trends), at the aggregate group level, at the community/family level, and at the species levels to detect any significant changes in value or composition over time, what the response times were, and whether these variables were resilient to the impacts or shifted to new conditions.

## Materials and methods

To determine the multi-scale effects of the 2014–2015 Pacific marine heatwave [33], distinct Gulf of Maine longer-term warming events (i.e., 2009–2011 temperature acceleration, 2012 marine heatwave, and post-2012 temperature spikes) [39, 40], and two Gulf of Mexico extreme events (i.e., Hurricane Katrina in 2005 and the Deepwater Horizon oil spill in 2010) [41, 42], we examined regional fisheries biomass [43] at the Large Marine Ecosystem (LME) scale [44], landings, and revenue datasets cumulatively and by functional group from several resources. U.S. fisheries landings data (1950–2019) from the Sea Around Us database at the LME scale were used as a proxy for fisheries biomass data [45], as applied in studies by Libralato et al. and Link [46, 47]. Data were obtained at the LME scale for areas encompassing the Alaskan region (i.e., “eastern Bering Sea” and “Gulf of Alaska” ecoregions), the Pacific northwest (U.S. component of the “Oregon, Washington, Vancouver Coast and Shelf” ecoregion), California (Northern California), northern Gulf of Mexico, and Gulf of Maine (U.S. component of the “Gulf of Maine/Bay of Fundy” ecoregion). These regions and LMEs have been described further by Sherman & Duda and Link & Marshak [48–50]. Data for all taxa were summed per year. Additionally, data from NOAA Fisheries fishery-independent groundfish bottom trawl surveys were used to ground-truth total biomass information from the Sea Around Us database and applied as a correction factor of magnitude between total values when applicable (i.e., dividing the total of each annual landing estimate from the Sea Around Us database by the corresponding annually calculated NOAA Fisheries survey biomass value [as published by Link & Marshak]) [49, 50], and dividing each annual total estimate by the total average quotient between both datasets for that region [51]. These corrections primarily were applied for the Gulf of Alaska and eastern Bering Sea regions. Corrections were applied in cases where magnitude between total survey biomass and totals from the Sea Around Us database did not align, as primarily observed for the Alaskan regions.

Fisheries landings and revenue data (1950–2021) for U.S. states encompassing the five regions of interest examined in this study were obtained from NOAA commercial and recreational landings databases [52]. Landings data from the Sea Around Us database at the LME scale, used as a proxy for biomass in this study, were found to be independent from NOAA U.S. landings data for each region in terms of their means as examined by t-test; distributions as examined by Kolmogorov-Smirnov testing; and time series as examined by cross-correlation tests (S1 Table). We chose to use the Sea Around Us data as a somewhat independent proxy for biomass to distinguish it from the more taxa specific estimates of trawl surveys to facilitate statistical assumptions of comparisons, and so that the results herein are comparable with prior studies. We included these data as an additional source of fisheries information at a different geographic scale and to prevent replication or pseudo-replication of fisheries data. Although Sea Around Us data are fishery-dependent information, our examinations and those from other studies find that they are independent from NOAA U.S. landings data given the distinct geographies at the LME scale and inclusion of foreign fishing data [46, 47]. Although some studies have pointed out the limitations of using fishery landings as a proxy for certain biomass trends [53], we reiterate that Sea Around Us data provided a proxy for fisheries biomass data in our study and as applied in other aforementioned cases [43, 44, 46, 47]. Data for these resources were acquired for all individual species occurring in those geographies, which also were summed per year. Annual Alaska landings and revenue are reported at the statewide level. Thus, values for these variables were estimated for the Gulf of Alaska and eastern Bering Sea by calculating annual Gulf of Alaska and eastern Bering Sea proportional landings contributions (i.e., fisheries biomass in this study) throughout the entire Alaskan region in the Sea Around Us database (i.e., at the species level) for a given year and then multiplying corresponding annual Alaska values for landings and revenue by those proportions.

Values for biomass, landings, and revenue were additionally categorized and summed in increasing hierarchical order, by family, as ratios of pelagic to demersal species (P/D ratios), as the percentage of shellfish (i.e., all bivalves and crustaceans), and at the total system level for each region. These variables may indicate differential responses within ecosystems and have been used in previous studies to examine effects to particular species groups from extreme events or predicted effects to functional groups from projected long-term changes in ocean conditions [54, 55]. Pelagic and demersal species were identified based on biological and life history classifications in the literature, local information from trawl surveys, and including web-based platforms (e.g., FishBase, SeaLifeBase) [56, 57]. Species classified as benthopelagic (e.g., Walleye pollock) were considered demersal species for this analysis. Eastern Bering Sea and Gulf of Alaska values at all resolutions also were estimated based on proportional contributions at the same resolution (i.e., by family, pelagic and demersal species, shellfish and non-shellfish species) as described above.

**Fig 1:**
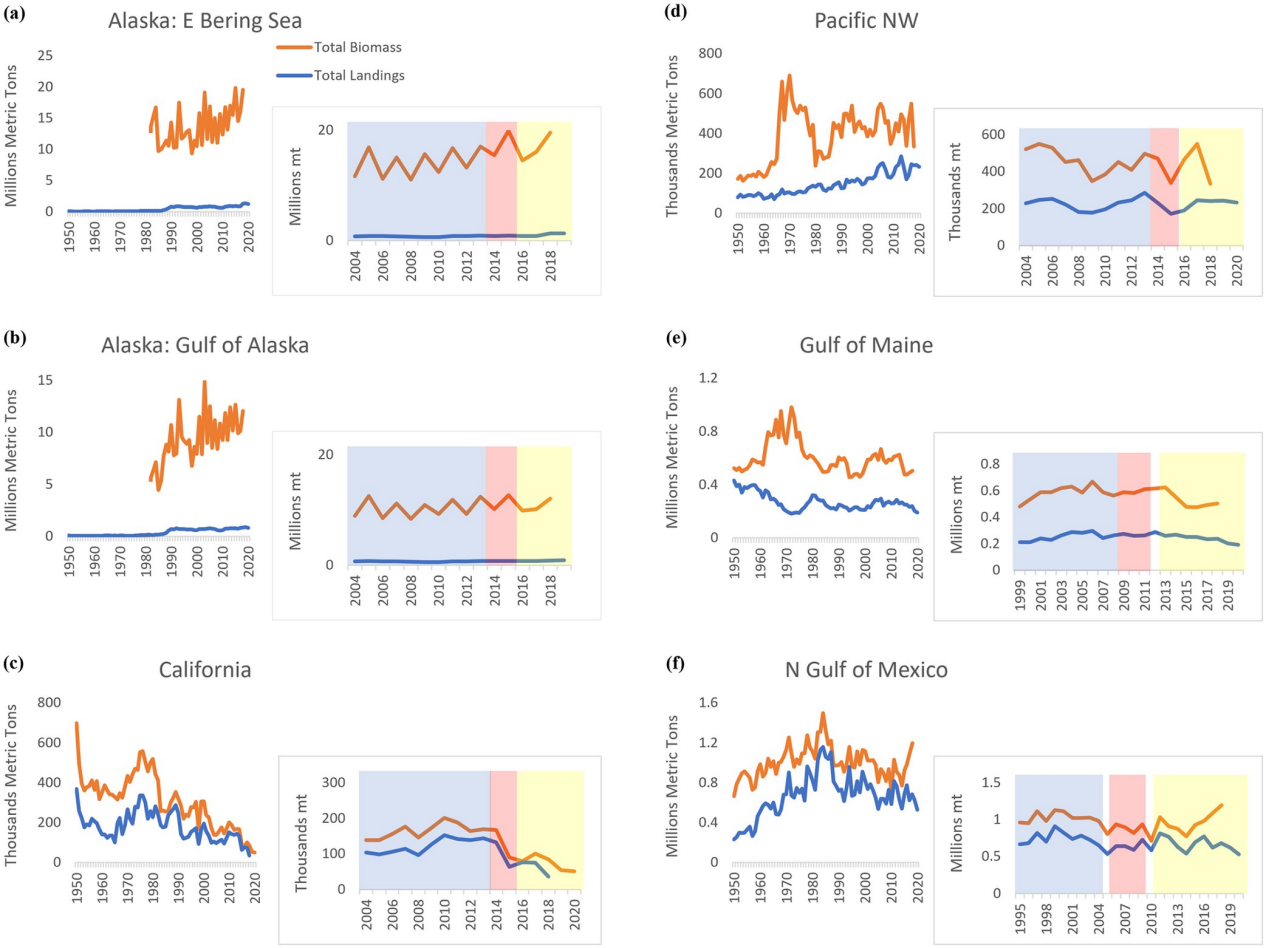
Total regional biomass and landings for fisheries ecosystems over time. For ecoregions investigated in this study (i.e., eastern Bering Sea, Gulf of Alaska, northern California, Pacific Northwest, Gulf of Maine, northern Gulf of Mexico), time series for total fisheries biomass and landings (1950–2020) are depicted. Additionally, for each region, biomass and landings trends for ten years pre-event (blue), during a marine heatwave or following an initial extreme event (pink), and post-heatwave or extreme event period (yellow) are shown. **(A)** For eastern Bering Sea, (**B**) Gulf of Alaska, **(C)** northern California, and **(D)** Pacific Northwest regions, these trends are shown ten years (2004–2013) prior to the Pacific marine heatwave (“Blob”), over the duration of the heatwave (2014–2015), and during years post-heatwave (2016–2020). **(E)** For the Gulf of Maine, they are shown ten years (1999–2008) prior to the onset of an accelerated warming period for the Gulf of Maine, during the accelerated warming period and prior to a subsequent marine heatwave and noted spike in temperatures (2009–2011), and for years following the 2012 heatwave and during the temperature spike (2013–2020). **(F)** For the northern Gulf of Mexico, trends are shown ten years (1995–2004) prior to Hurricane Katrina, in years post-hurricane and prior to the Deepwater Horizon (DWH) oil spill (2006–2009), and for years following the DWH event (2011–2020).

**Fig 2:**
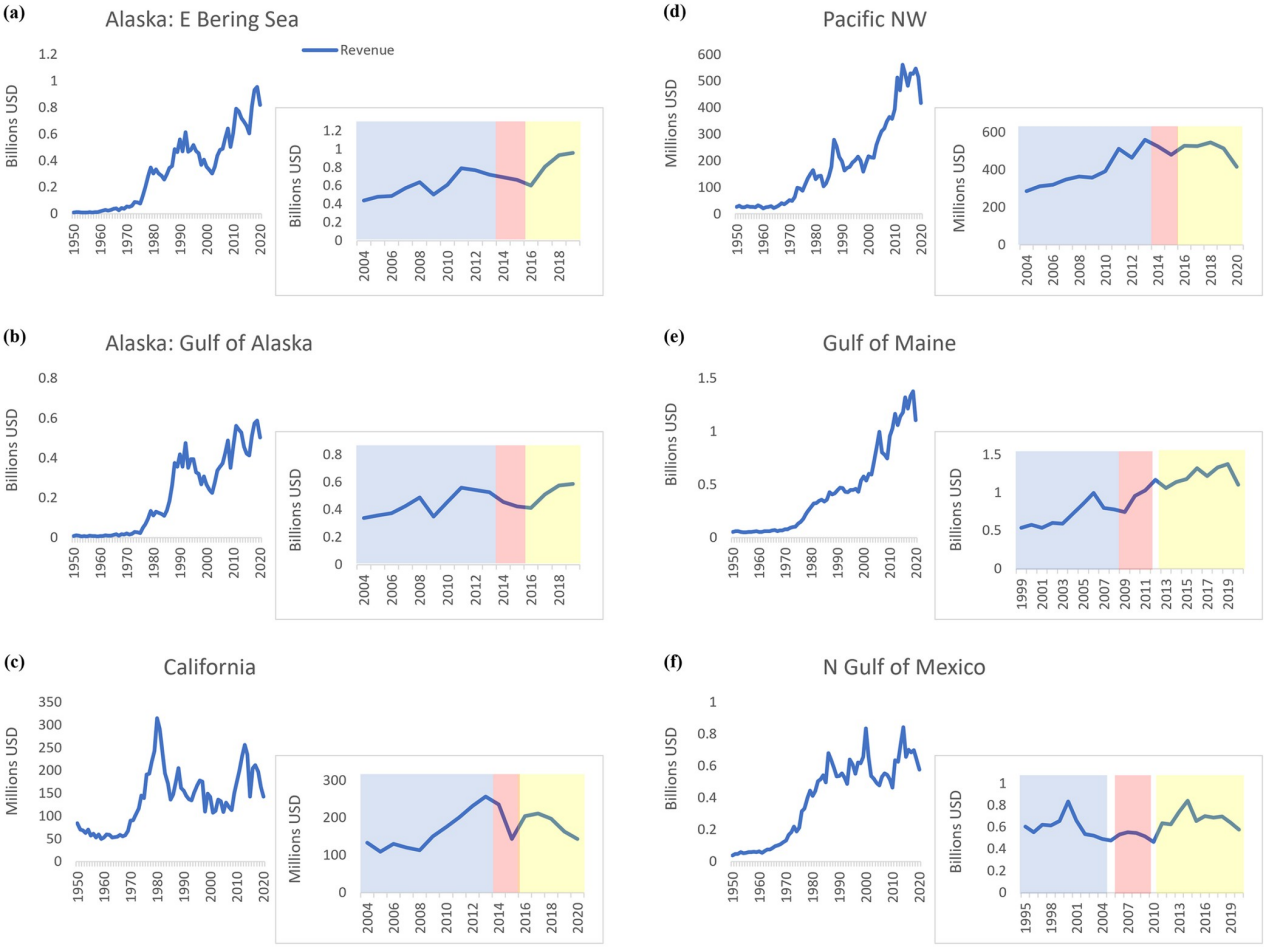
Total regional revenue for examined fisheries ecosystems over time. For ecoregions (i.e., eastern Bering Sea, Gulf of Alaska, northern California, Pacific Northwest, Gulf of Maine, northern Gulf of Mexico), time series for total fisheries revenue (1950–2020) are shown. Additionally, for each region, biomass and landings trends for ten years pre-event (blue), during a marine heatwave or following an initial extreme event (pink), and post-heatwave or extreme event period (yellow) are depicted. **(A)** For eastern Bering Sea, **(B)** Gulf of Alaska, **(C)** northern California, and **(D)** Pacific Northwest regions, trends in revenue are shown ten years (2004–2013) prior to the Pacific marine heatwave (“Blob”), over the duration of the heatwave (2014–2015), and during years post-heatwave (2016–2020). **(E)** For the Gulf of Maine, they are shown ten years (1999–2008) prior to the onset of an accelerated warming period for the Gulf of Maine, during the accelerated warming period and prior to a subsequent marine heatwave and noted spike in temperatures (2009–2011), and for years following the 2012 heatwave and during the temperature spike (2013–2020). **(F)** For the northern Gulf of Mexico, revenue trends are shown ten years (1995–2004) prior to Hurricane Katrina, in years post-hurricane and prior to the Deepwater Horizon (DWH) oil spill (2006–2009), and for years following the DWH event (2011–2020).

Trends for fisheries biomass, landings, and revenue were examined over time, and among distinct periods (see Figs 1 and 2 and Tables 1 and 2 for specific timeframes) to quantify the effects of particular extreme events on those variables for a given ecosystem. Periods of interest included the cumulative ten years prior to a given event (i.e., Pacific marine heatwave, accelerated warming period and subsequent marine heatwave in the Gulf of Maine, Hurricane Katrina in the northern Gulf of Mexico), the time over which a given event occurred, and the subsequent years following a given event (and/or prior to another event in the case of the Deepwater Horizon oil spill in the Gulf of Mexico or post-heatwave observed spikes in temperatures for the Gulf of Maine). These time periods were classified as occurring “pre-event”, “during event”, and “post-event.” Trends within and among distinct time periods were examined for biomass, landings and revenue for the species, family, P/D ratio, percentage of shellfish, and total system level over time.

**Table 1:** Analysis of variance results for total biomass, landings, and revenue values per examined fisheries ecosystem over time.

Region	Total		Pelagic		Demersal		Shellfish		Non-Shellfish	
*Biomass*	**F**	**P**	**F**	**P**	**F**	**P**	**F**	**P**	**F**	**P**
E Bering Sea	2.432	0.1299	6.734	0.0109*	4.581	0.0333*	3.104	0.0819	4.219	0.0410*
Gulf of Alaska	0.4229	0.6645	7.469	0.0078**	7.469	0.0078**	3.831	0.0517	3.831	0.0517
California	22.3	<0.0001***	22.62	<0.0001***	0.2661	0.7702	0.8854	0.9159	24.2	<0.0001***
Gulf of Maine	3.543	0.0533	1.578	0.2368	3.622	0.0504	1.472	0.259	1.211	0.3238
Gulf of Mexico	2.928	0.0779	2.731	0.0907	17.39	<0.0001***	0.4898	0.6203	4.007	0.0353*
Pacific NW	0.453	0.6462	0.4641	0.6395	1.65	0.2328	4.701	0.0311*	0.6926	0.5192
*Landings*	**F**	**P**	**F**	**P**	**F**	**P**	**F**	**P**	**F**	**P**
E Bering Sea	6.237	0.0126*	1.625	0.2345	7.595	0.0065**	2.598	0.1124	7.814	0.0059**
Gulf of Alaska	4.535	0.0321*	1.627	0.2342	5.934	0.0148*	6.151	0.0132*	6.114	0.0134*
California	6.855	0.0103*	6.812	0.0106**	1.271	0.3158	0.1996	0.822	7.874	0.0065**
Gulf of Maine	1.338	0.2873	3.398	0.0560	1.632	0.2232	29.99	<0.0001***	9.617	0.0014**
Gulf of Mexico	3.137	0.0643	1.99	0.1616	11.42	0.0004***	8.641	0.0018**	2.993	0.0718
Pacific NW	0.6737	0.5256	0.3399	0.7175	1.472	0.2629	2.021	0.1694	1.401	0.2789
*Revenue*	**F**	**P**	**F**	**P**	**F**	**P**	**F**	**P**	**F**	**P**
E Bering Sea	4.165	0.04*	0.6782	0.5246	3.487	0.0613	0.977	0.4024	4.151	0.0404*
Gulf of Alaska	1.483	0.263	0.6825	0.5226	2.583	0.1136	0.4858	0.6259	3.146	0.0769
California	0.4978	0.6182	0.3904	0.6839	2.193	0.1485	0.7822	0.4764	0.2846	0.757
Gulf of Maine	30.11	<0.0001***	0.0711	0.9316	37.34	<0.0001***	39.72	<0.0001***	0.9604	0.4015
Gulf of Mexico	4.937	0.0175*	14.63	0.0001***	2.473	0.1085	1.292	0.2956	28.79	<0.0001***
Pacific NW	4.313	0.0347*	0.0179	0.9823	6.362	0.0108*	11.59	0.0011**	1.657	0.2261

For each ecosystem (i.e., eastern Bering Sea, Gulf of Alaska, northern California, Pacific Northwest, Gulf of Maine, northern Gulf of Mexico), F and p-values provided for each analysis of variance model to examine significant differences in total biomass, landings, and revenue among periods (i.e., pre-event, event, and post-event), including pelagic and demersal and shellfish (i.e., crustaceans and bivalves) and non-shellfish components, associated with each variable over time. For eastern Bering Sea, Gulf of Alaska, northern California, and Pacific Northwest regions, tests of significance in values were conducted for time periods ten years prior to the Pacific marine heatwave (“Blob”), over the duration of the heatwave, and post-heatwave. For the Gulf of Maine, differences in values were examined among time periods ten years prior to the onset of an accelerated warming period for the Gulf of Maine, during the accelerated warming period and prior to a subsequent marine heatwave and noted spike in temperatures, and for years following the heatwave and during the temperature spike. For the northern Gulf of Mexico, tests were conducted for time periods ten years prior to Hurricane Katrina, during the post-hurricane period prior to the Deepwater Horizon (DWH) oil spill, and post-DWH event. Bold values indicate statistically significant relationships (*p≤0.05; **p≤0.01; ***p≤0.001). Multivariate analysis of variance (MANOVA) information is provided in S2 Table.

**Table 2:** Analysis of similarity for total biomass, landings, and revenue values per examined fisheries ecosystem over time.

	Pre-Heatwave v. Heatwave				Pre-Heatwave v. Post-Heatwave				Heatwave v. Post-Heatwave			
Pacific Ecosystems	E Bering Sea	Gulf of Alaska	California	Pacific NW	E Bering Sea	Gulf of Alaska	California	Pacific NW	E Bering Sea	Gulf of Alaska	California	Pacific NW
Biomass	0.6648	0.6781	0.0453*	0.0947	0.0942	0.1756	0.0004***	0.0215*	0.5944	0.594	0.236	0.7983
Landings	0.7934	0.8179	0.1229	0.0589	0.0203*	0.1095	0.004**	0.0009***	0.1306	0.2003	0.801	0.0473*
Revenue	0.276	0.2659	0.1159	0.0614	0.0358*	0.0066**	0.0075**	0.0033**	0.7281	0.6646	0.2329	0.0940
**Gulf of Maine**	**Pre-Accel v. Post-Accel/Post-Heatwave**				**Pre-Accel v. Accel/Pre-Heatwave**				**Post-Accel/Post-Heatwave v. Accel/Pre-Heatwave**			
Biomass	0.0003***				0.1009				0.1207			
Landings	0.0001***				0.0134*				0.042*			
Revenue	0.0001***				0.1056				0.0048**			
**Gulf of Mexico**	**Pre-Katrina v. Post-Katrina / Pre-DWH**				**Pre-Katrina v. Post-DWH**				**Post-Katrina / Pre-DWH v. Post-DWH**			
Biomass	0.0405*				0.0002***				0.1968			
Landings	0.052				0.0172*				0.409			
Revenue	0.0051**				0.0001***				0.0016**			

For each ecoregion (i.e., eastern Bering Sea, Gulf of Alaska, northern California, Pacific Northwest, Gulf of Maine, northern Gulf of Mexico), p-values provided for each analysis of similarity (ANOSIM) test to examine significant differences in species composition for biomass, landings, and revenue among pre-event, event, and post-event periods. For eastern Bering Sea, Gulf of Alaska, northern California, and Pacific Northwest regions, tests of significance in values were conducted for time periods ten years prior to the Pacific marine heatwave (“Blob”), over the duration of the heatwave, and post-heatwave. For the Gulf of Maine, differences in values were examined among time periods ten years prior to the onset of an accelerated warming period for the Gulf of Maine, during the accelerated warming period and prior to a subsequent marine heatwave and noted spike in temperatures, and for years following the heatwave and during the temperature spike. For the northern Gulf of Mexico, tests were conducted for time periods ten years prior to Hurricane Katrina, during the post-hurricane period prior to the Deepwater Horizon (DWH) oil spill, and post-DWH event. Bold values indicate statistically significant relationships (*p≤0.05; **p≤0.01; ***p≤0.001). Additional information (i.e., ANOSIM global R and global p-values) is provided in S4 Table.

Significant differences in biomass, landings, and revenue, at the various taxonomic levels or aggregations (i.e., annual totals; annual pelagic, demersal, shellfish, and non-shellfish biomass, landings, and revenue, and/or ratios and percentages thereof) within a given ecoregion were explored through Multivariate Analysis of Variance (MANOVA), univariate Analysis of Variance (ANOVA), and post-hoc pairwise comparisons (i.e., Tukey tests) for each of those variables among time periods of interest. All values were examined using Levene’s tests to confirm assumptions for parametric testing. Percentage values were arcsine square root transformed in accordance with assumptions of normality for parametric testing. These ratios and percentages also were examined across all regions to determine their influence on total system-level trends. Additionally, any differences in species composition among time periods of interest were examined through Analysis of Similarity testing (ANOSIM; Bray-Curtis dissimilarity matrix), including Similarity Percentages (SIMPER) testing to quantify which species were the greatest contributors to any differences among time periods. Trends in the abundance or value of dominant families for a given ecoregion (i.e., top ten most abundant over timeframe of interest) among time periods also were examined to describe dynamics occurring at that scale for fisheries biomass, landings, and revenue.

## Results

### Pacific marine heatwave event

Total fisheries biomass and landings (Fig 1a–1d) exhibited differential trends in U.S. west coast ecosystems affected by the Pacific marine heatwave (“Blob”). Major increases in eastern Bering Sea and Gulf of Alaska estimated landings occurred during the 1980s, with values currently peaking at ~1.34 million mt and 896.5 thousand mt, respectively. Regular annual surveys of fisheries biomass since the 1980s have shown an increasing trend, with values generally around 10.5–19.9 million mt in the eastern Bering Sea and 7.9–12.1 million mt in the Gulf of Alaska since the 2000s. Over the past 70 years, total biomass and landings in northern California have decreased, especially since the 1980s, each with significant declines (Table 1; S1a Fig) occurring between pre-event (years 2004–2013) and post-event (years 2016–2020) periods. Values for both biomass and landings are currently ~50–100 thousand mt. In the Pacific northwest (PNW), total biomass has oscillated with major increases observed during the 1960s-1970s, a subsequent decline to around 200 thousand mt in the 1980s, and values remaining generally stable around 300–500 thousand mt since the 1990s. PNW landings have generally increased over time, averaging around 200 thousand mt since the mid-2000s. No significant difference in PNW total biomass or landings occurred during time periods associated with the heatwave.

No significant difference in total eastern Bering Sea or Gulf of Alaska biomass was observed among heatwave time periods, while a significant increase between pre-heatwave and post-heatwave periods was found for total estimated eastern Bering Sea and Gulf of Alaska landings. Overall, trends in total biomass and landings during Pacific heatwave periods were generally stable, with the greatest exceptions observed for the California ecosystem, particularly for pelagic (S2a Fig) and non-shellfish biomass and landings (S3a Fig). Following the heatwave, significant increases in eastern Bering Sea and Gulf of Alaska demersal biomass, demersal landings, and non-shellfish landings were observed compared to pre-heatwave values, as was a significant increase in eastern Bering Sea non-shellfish biomass. Significant decreases in eastern Bering Sea and Gulf of Alaska pelagic biomass, and Gulf of Alaska shellfish landings also occurred between those periods. Shellfish and pelagic components contribute smaller proportions of Alaskan biomass and landings. Additionally, significant increases in total fisheries revenue (Fig 2), and demersal and shellfish revenue, were only observed among Pacific heatwave periods for the eastern Bering Sea and PNW. Generally, over longer timeframes, revenue has increased for the eastern Bering Sea and Gulf of Alaska regions to ~958 million and 588 million USD, respectively, and up to ~500 million USD in the PNW, while values for California have oscillated with a peak ~300 million USD in the early 1980s, followed by lower values (~150 million USD), and later increases to ~200–250 million USD as of the 2010s. Significant relationships were observed among all variables for California and PNW ecosystems between pre-heatwave and post-heatwave periods (S2 Table).

**Fig 3:**
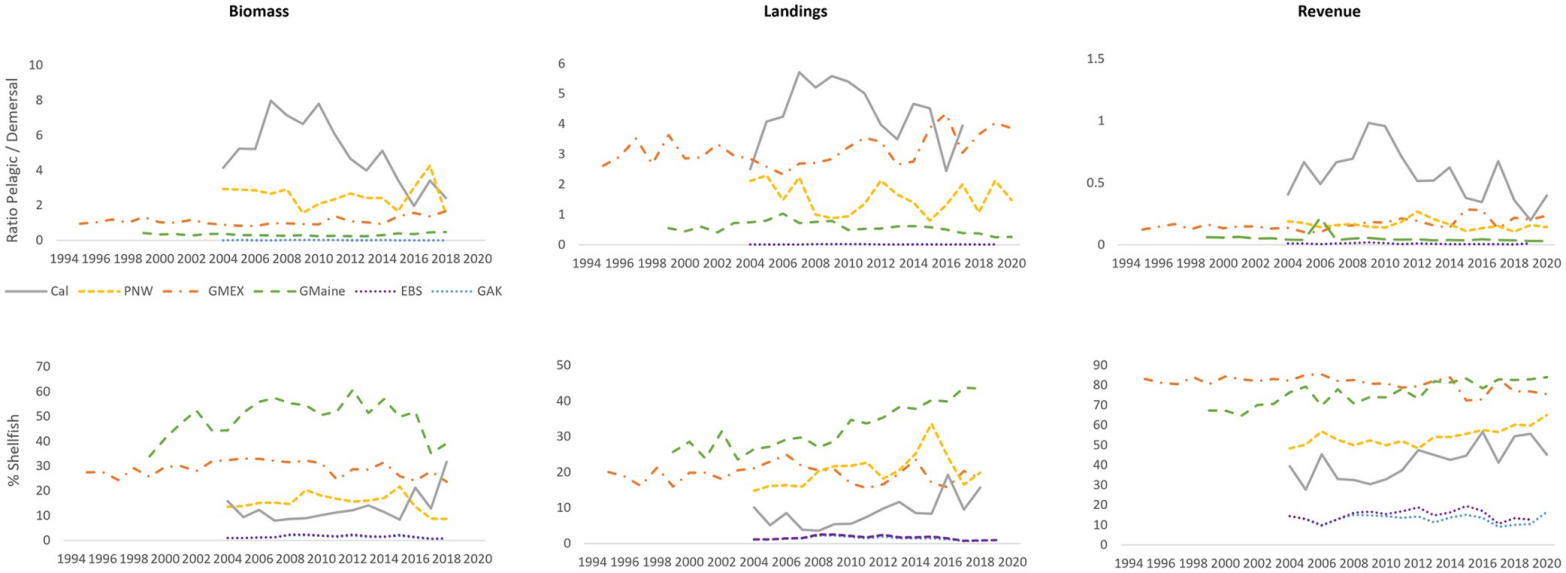
Pelagic to demersal ratios and percent contribution of shellfish (% shellfish) per fisheries ecosystem over time. For each fisheries ecosystem (i.e., eastern Bering Sea–EBS; Gulf of Alaska–GAK; Northern California–Cal; Pacific Northwest–PNW; Gulf of Maine–GMaine; Northern Gulf of Mexico–GMEX), pelagic to demersal ratios for (top panel) and percent contribution of total shellfish (i.e., bivalves and crustaceans; bottom panels) to total biomass, landings, and revenue are shown over time. For eastern Bering Sea, Gulf of Alaska, northern California, and Pacific Northwest ecosystems, values are shown for 2004–2020 (i.e., over time periods ten years prior to the Pacific marine heatwave (“Blob”), over the duration of the heatwave, and post-heatwave). For the Gulf of Maine, values are depicted for 1999–2020 (i.e., over time periods ten years prior to the onset of an accelerated warming period for the Gulf of Maine, during the accelerated warming period and prior to a subsequent marine heatwave and noted spike in temperatures, and for years following the heatwave and during the temperature spike). For the northern Gulf of Mexico, values are shown for 1995–2020 (i.e., over time periods ten years prior to Hurricane Katrina, during the post-hurricane period prior to the Deepwater Horizon (DWH) oil spill, and for years following the DWH event).

Generally, significant relationships among pelagic to demersal (P/D) ratios and among percent shellfish of biomass, landings, and revenue were observed in all systems except for PNW pelagic to demersal ratios, and for all eastern Bering Sea percent shellfish values (S3a Table). Among all regions, P/D ratios generally were largest for California biomass, landings, and revenue, and secondarily for PNW biomass. Additionally, annual percentages of shellfish were generally higher in California and PNW biomass, landings, and revenue. Significant decreases in P/D ratios were observed in biomass for the eastern Bering Sea, Gulf of Alaska, and California ecosystems between pre-heatwave and post-heatwave periods (S3b Table; Fig 3). Significant increases in the percentage of shellfish between pre-heatwave and post-heatwave periods occurred for California biomass, California revenue, and PNW revenue. Increases also occurred for the percentage of shellfish in Gulf of Alaska biomass and landings between pre-heatwave and post-heatwave periods and between heatwave and post-heatwave periods. The percentage of shellfish in PNW biomass significantly decreased between pre-heatwave and post-heatwave periods. For PNW landings, a significant increase occurred between pre-heatwave and heatwave periods, which was followed by a significant decrease post-heatwave to within pre-heatwave values.

Significant differences in species composition for total biomass, landings, and revenue (Table 2; S4 Table) were observed across heatwave-related periods for Pacific ecosystems. Generally, differences mostly occurred between pre-heatwave and post-heatwave periods for all three regions, with shifts in biomass and landings composition observed both for California and PNW ecosystems. Changes in the composition of eastern Bering Sea landings also were observed between those periods, but were minimal in the Gulf of Alaska. Significant shifts in revenue composition also were observed in all four regions between pre- and post-heatwave timeframes. Pre-heatwave and post-heatwaves changes in California biomass and landings composition were most related to declines in California market squid (*Doryteuthis opalescens*; family Loliginidae) and Pacific sardine (*Sardinops sagax*; family Clupeidae) biomass and landings, and increases in Northern anchovy (*Engraulis mordax*; family Engraulidae) biomass (S5 Table; S4a Fig). A significant shift in California biomass composition also was observed between pre-heatwave and heatwave periods, with changes in the same three species being top contributors, and with similar contributions by other major species, for which decreases were observed (e.g., Dungeness crab—*Metacarcinus magister*, family Cancridae; Pacific hake—*Merluccius productus*) An increase in Ocean shrimp (*Pandalus jordani*) biomass also occurred between pre-heatwave and heatwave periods. The greatest contributors to pre-heatwave and post-heatwave shifts in California revenue were California market squid, for which a decrease was observed, and for Dungeness crab and Pacific oyster (*Crassostrea gigas)*, whose revenue increased between these periods. Additionally, increases in revenue for crustaceans were observed post-heatwave.

Changes in PNW biomass were most related to increases in Pacific hake (family Merluccidae), declines in Pacific sardine (family Clupeidae) and Pacific oyster, and increases in Sockeye salmon (*Oncorhynchus nerka*; family Salmonidae) (S5 Table, S4a Fig). Similarly, these trends also were observed for Pacific hake and Pacific sardine landings between pre- and post-heatwave periods, with additional shifts in Ocean shrimp (i.e., an increase in landings between pre-heatwave and post-heatwave periods; Table 2; S5 Table; S4a). Likewise, these three species were the greatest contributors to shifts in PNW landings between heatwave and post-heatwave timeframes, with increases observed for Pacific hake and decreases in Ocean shrimp between these periods. Changes in PNW revenue between pre- and post-heatwave periods were predominantly due to increases for Dungeness crab, Pacific geoduck clam (*Panopea generosa*), and Pacific oyster, and Ocean shrimp.

No significant differences in species composition were observed for Alaskan biomass among time periods (Table 2; S1a and S4a Figs), while significant changes in eastern Bering Sea landings primarily were due to increases in Walleye pollock (*Gadus chalcogrammus*; family Gadidae), Sockeye salmon, Pacific cod (*Gadus macrocephalus*; family Gadidae), and slight decreases in Pink salmon (*O*. *gorbuscha*; family Salmonidae). Additionally, significant pre- and post-heatwave shifts in eastern Bering Sea and Gulf of Alaska revenue were most related to increases in Walleye pollock, and Sockeye and Pink salmon, and decreases in Pacific halibut (*Hippoglossus stenolepis*; family Pleuronectidae). Among regions, some increases in biomass, landings, and revenue between periods were observed for other species with lower contributions to composition shifts (S5 Table), although at varying magnitudes.

### Atlantic events—Gulf of Maine warming and northern Gulf of Mexico Hurricane Katrina and Deepwater Horizon (DWH)

Total biomass and landings in the Gulf of Maine have fluctuated and generally decreased over the past 70 years, with pronounced declines in biomass occurring during the 1970s and remaining at ~500–700 thousand mt in subsequent years (Fig 1e). Reductions in landings were observed from the 1950s-1960s, with some increases occurring in the 1970s and values remaining generally stable from the 1980s onward. No significant differences in total biomass or total landings were observed among time periods of interest (1999–2020) (Table 1; S1b Fig). These non-significant trends also were observed for pelagic and demersal biomass and landings (S2b Fig). Significant increases in Gulf of Maine shellfish landings occurred over the time periods of interest (S3b Fig). Whereas, a significant decrease in non-shellfish landings was observed between pre- and post-acceleration periods. Total fisheries revenue in the Gulf of Maine has increased to >1 billion USD over time, with steep intensification in value since the late 1990s (Fig 2e). Significant increases in total revenue were detected among all three warming-related periods of interest over the 21-year timeframe (Table 1), including for demersal and shellfish revenue. Significant relationships also were observed among all variables in the Gulf of Maine ecosystem during these time periods (S2 Table).

Significant relationships were observed among Gulf of Maine percent shellfish of biomass, landings, and revenue (S3a Table). A significant decrease in P/D ratios for Gulf of Maine landings was observed following the 2009–2011 temperature acceleration and 2012 heatwave (S3b Table; Fig 3). Significant increases were observed for percent shellfish landings and revenue during the post-acceleration/post-heatwave period to levels that were significantly higher than pre-acceleration values. A significant increase in percent shellfish landings occurred between the pre-acceleration and acceleration/pre-heatwave time periods as well. Pelagic/demersal ratios in the Gulf of Maine were comparatively lower over time than for other regions. However, the percentage of shellfish for Gulf of Maine biomass and landings throughout the period of study, and for revenue post-2014, was comparatively greatest over time.

Significant differences in species composition for total biomass, landings, and revenue were observed among pre-acceleration, acceleration/pre-heatwave, and post-acceleration/post-heatwave periods in the Gulf of Maine (Table 2; S4 Table). Differences were detected for total landings across all three timeframes. Changes in biomass between the pre-acceleration and post-acceleration/post-heatwave periods were most related to decreases in American sea scallop (*Placopecten magellanicus*), and increases in Atlantic surf calm (*Spisula solidissima*), Ocean quahog (*Arctica islandica*), and American lobster (*Homarus americanus*). Changes in landings also were most associated with those species, as well as Atlantic herring (*Clupea harangus*; family Clupeidae), Atlantic mackerel (*Scomber scombrus*; family Scombridae), and Atlantic cod (*Gadus morhua*; family Gadidae; S5 Table; S4b Fig). Trends in landings for these species differed between time periods, with their increases most observed between the pre-acceleration and acceleration/pre-heatwave periods and pre-acceleration and post-acceleration/post-heatwave periods.

Increases in cumulative bivalve biomass were observed between pre- and post-acceleration/post-heatwave periods, with decreases to lower than pre-acceleration values following the 2012 heatwave (S4b Fig). Cumulative bivalve landings marginally increased during the post-acceleration/post-heatwave period and generally remained stable. Little changes in cumulative herring biomass were observed among timeframes, although landings decreased with lowest values post-acceleration/post-heatwave. Increases in lobster biomass and particularly landings occurred during the post-acceleration/post-heatwave period, while decreases in cod (family Gadidae) landings were observed during this time period. Changes in revenue among time periods were most related to those for all aforementioned species, especially increases for American sea scallop, American lobster, Atlantic cod, and decreases for Atlantic herring. Lobster and bivalve revenue consistently increased throughout warming periods to near 600 million and over 400 million USD, respectively. Since 1950, Gulf of Mexico fisheries biomass and landings have followed similar patterns as to the Gulf of Maine, with increasing trends in earlier decades, and highest values observed in the early-to-mid-1980s prior to decreases and more stable values in more recent years (Fig 1f). Following their peak values (biomass: ~1.5 million mt; landings ~1.1 million mt), total biomass and landings in the Gulf of Mexico have averaged ~970 thousand mt and ~700 thousand mt, respectively, since 1990. No significant differences in total Gulf of Mexico biomass and landings were observed among time periods (Table 1; S1b Fig). However, a significant decrease in demersal biomass from earlier values occurred during the post-Katrina/post-DWH period (S2b Fig). A significant decrease in non-shellfish biomass also was observed during the post-Katrina/pre-DWH period, with no significant changes from earlier values observed afterward (S3b Fig). Furthermore, significant decreases in demersal and shellfish landings from earlier values occurred during the post-Katrina/post-DWH period. Total fisheries revenue in the Gulf of Mexico has been variable, with increases up to ~600 million USD in the mid-1980s and oscillating values in years since. Peak values of ~850 million USD occurred in both 2000 and 2014, with total revenue averaging ~560 million USD in intermediate years, and declines to ~660 million USD observed in more recent years (Fig 2f). A significant decrease in revenue was observed between the pre-Katrina and post-Katrina/pre-DWH periods, with post-Katrina/post-DWH revenue having returned to values similar to those observed in the pre-Katrina period. Additionally, significant increases in pelagic and non-shellfish revenue were observed during the post-Katrina/post-DWH period as compared to earlier values. Significant relationships also were observed among all variables in the Gulf of Mexico ecosystem during these time periods (S2 Table).

Significant relationships were observed among Gulf of Mexico P/D ratios and among percent shellfish of biomass, landings, and revenue (S3a Table). Significant increases in P/D ratios for Gulf of Mexico biomass and landings occurred between the post-Katrina/pre-DWH and post-Katrina/post-DWH periods (S3b Table; Fig 3). Significant decreases in percent shellfish biomass, landings, and revenue also were observed between these periods. Additionally, a significant increase in P/D ratios for revenue was observed between the pre-Katrina and post-Katrina/post-DWH periods, while a concurrent decrease in percent shellfish revenue also occurred between these periods. Among all regions and variables, pelagic/demersal ratios for the Gulf of Mexico were comparatively second- (landings, revenue) or third-highest (biomass) over time, while the percentage of shellfish in Gulf of Mexico biomass and landings was comparatively second-highest, and until recent years was highest for revenue.

Significant differences in species composition for total biomass, landings, and revenue were observed among distinct time periods (Table 2; S4 Table). Differences for all variables were observed between pre-Katrina and post-Katrina/post-DWH periods. Between these timeframes, changes in biomass were most associated with decreases in the abundance of Gulf menhaden (*Brevoortia patronus*; family Clupeidae), Eastern oyster (*Crassostrea virginica*), and Atlantic croaker (*Micropogonias undulatus*; family Sciaenidae), and increases in White shrimp (*Litopenaeus setiferus*). Additionally, shifts in menhadens (*Brevoortia* spp.), White and Brown shrimp (*Farfantepenaeus aztecus*), Red drum (*Sciaenops ocellatus*), and Spotted seatrout (*Cynoscion nebulosus*) contributed most to changes in landings (S5 Table; S4b Fig). Additional differences were observed for biomass composition between the pre-Katrina and post-Katrina/pre-DWH periods, with greatest contributors including all aforementioned species for biomass and miscellaneous crustaceans. Decreases in herring biomass and landings were observed between the pre-Katrina and post-Katrina periods, with increases observed to near pre-Katrina values in post-Katrina/post-DWH years (S4b Fig). Less pronounced decreases over time also were observed for bivalve and drum (Family Sciaenidae) biomass. Additionally, marginal decreases in shrimp landings were observed among time periods, primarily for Brown shrimp.

Pre-Katrina and post-Katrina/post-DWH changes in revenue were most related to shifts in the values of Brown shrimp, menhadens, White shrimp, Eastern oyster, Blue crab (*Callinectes sapidus*), and crayfishes (*Procambarus* spp.; S5 Table; S4b Fig). Revenue values for most of these species, except Brown shrimp, increased between time periods. Significant differences in revenue composition were observed among all time periods, with changes between the pre-Katrina and post-Katrina/pre-DWH periods most related to shifts in the above species, and those between the post-Katrina/pre-DWH and post-Katrina/post-DWH timeframes related to aforementioned species and Red snapper (*Lutjanus campechanus*). Decreases in shrimp revenue (primarily for Brown shrimp) occurred in periods before and after Hurricane Katrina, with some rebound observed post-Katrina and DWH. While comparatively lower, increases in revenue for herrings, bivalves, and crabs also occurred among time periods, particularly in more recent years post-DWH.

## Discussion

There were clear responses to extreme events and distinct warming periods that were detectable in the marine ecosystems that we examined in terms of differences for biomass, landings, and revenue among time periods. They were observed across multiple facets of these socio-ecological systems. The responses varied, but that we were readily able to detect these responses in association with extreme events is not trivial. Higher levels of the biological hierarchy in general tended to be more stable than lower levels, particularly when compared to the species level. This pattern varied across ecosystems, but generally the increasing number of significant responses as one moves down the biological hierarchy was commonly observed, consistent with what one would similarly expect from hierarchy theory [13, 58–60]. Greater stability at the system-level generally occurred for all three response variables, and demonstrates the stability expected at higher levels of aggregation [60–62]. Although additional compounding and confounding factors may also influence these ecosystem responses, the findings in this study generally confirm the value of understanding emergent outcomes at multiple scales of hierarchy.

The most pronounced multi-level responses occurred in the California ecosystem, for which significant decreases in biomass and landings were observed post-event at the system level and for specific species and species complexes. These decreases appear to be related to the greater pelagic composition of this system, including the importance of squid, sardine, and anchovy that are responsive to climatic changes and more sensitive to thermal dynamics occurring in the water column [33, 62–65]. Previous studies have documented these decreases, including pronounced declines in pelagic, nearshore, and larval fishes over time [66] and dominance of particular taxa (e.g., Pacific sardine or northern anchovy) during varying periods. These factors may explain why biomass and landings in this system (and in the Gulf of Mexico where Gulf Menhaden is a dominant component of biomass and landings) tracked more closely than in other ecosystems. Additional studies observed a northward migration of squid out of the California region during the “Blob,” lower recruitment for Pacific sardine, and reduced nutrient content for key forage species, which contributed to the altered composition of this system to one with increasing proportions of crustaceans, bivalves, and other demersal species [33, 62]. Similar decreases in herrings and other pelagic species were observed in Gulf of Alaska and eastern Bering Sea biomass post-heatwave, as observed in additional studies of Alaska in which forage fish became scarce in response to regional warming [67–69]. Furthermore, other studies observed post-heatwave oscillations in eastern Bering Sea zooplankton, including in response to lingering effects and subsequent heatwaves in that region, which also may have affected pelagic biomass [70–72]. Decreases in Gulf of Maine herrings also were observed in our study during the post-acceleration/post-heatwave period, illustrating similar warming effects to herrings across several regions and suggesting differential warming effects depending on pelagic and demersal composition of a given ecosystem. Despite similar responses, it is possible that changes in herring abundance in these systems may have been driven by one or more other forcing factors. For example, periodic increases in herring abundance have been observed in the eastern Bering Sea in response to more recent warming periods, suggesting the influences of additional factors [72]. The duration of warming periods and functional similarity of certain ecosystems (e.g., abundance of Pacific sardines in Gulf of Alaska and California systems) also suggest broader influences leading to these responses. However, collectively it does appear that those ecosystems with a higher proportion of pelagic biomass tend to exhibit stronger and more widespread responses to extreme temperature events. Observed redistributions in U.S. pelagic fishing fleets following marine heatwaves appear to support this observation [73].

Although effects to Dungeness crabs were inferred in this study (i.e., contributed to changes in biomass, landings, and revenue among regions), and in others that have evaluated effects from the “Blob” [15, 33, 74], the continued persistence of other demersal shellfish species and fishery diversification appears to contribute toward economic resilience at the system level, as also observed in this study for the Gulf of Alaska and the PNW. Similar trends for crustaceans and for bivalve landings and revenue occurred in the Gulf of Maine, with declines in small pelagic and some demersal fishes mostly occurring during the post-acceleration/post-heatwave period. Studies have postulated that proactive management efforts helping to mitigate effects of warming-associated early lobster migrations on crustacean fisheries, and differential vulnerability of bivalve fisheries and their revenue to marine heatwave effects, may contribute to this observed resilience [15, 33, 74, 75]. Additionally, adaptations by some fishing communities in response to extreme event-driven declines in target finfish species may contribute to the increase in shellfish landings and their revenues [76–78], but also may come with associated risks in some cases [79]. Further, declines in Atlantic cod and other groundfish may have allowed for predatory release on lobsters and other invertebrates, potentially contributing to some of this resilience [76, 80, 81]. However, other systems, such as the EBS, have observed pronounced declines in snow crab and certain other crustaceans following warming periods [82], indicating differential ecological and physiological responses by shellfish and non-shellfish species among systems that may affect ecosystem composition. Recent examinations also have illustrated greater potential resilience in demersal fish biomass in response to marine heatwaves [83]. Furthermore, climate vulnerability assessments for California Current species suggest that species with longer lifespans, habitat specialization, and low population growth rates are most vulnerable to regional warming effects [84]; however, inconsistencies also have been observed in responses of sessile animals and their recruits to marine heatwaves [15, 85, 86]. Additionally, differential effects from bottom marine heatwaves, which have affected these ecosystems over time, may compound ecological and socioeconomic responses with varying degrees of resilience to these events [68, 69, 72, 87]. Furthermore, heatwaves have continued along the U.S. Pacific coast in more recent years since the “Blob” (i.e., 2019, 2020), with impacts to and displacements of top predator distributions [88]. These observations reinforce the differential vulnerabilities and resiliencies of particular species groups to extreme events, despite greater potential resilience generally observed at the system level.

Conversely, mixed responses to extreme events occurred in the Gulf of Mexico, where changes in demersal biomass and shrimp landings were observed, while increases in pelagic and non-shellfish revenue occurred following the DWH event. Granted, the nature of the extreme events in the Gulf of Mexico were of a different nature than warming or heatwaves experienced in marine ecosystems elsewhere in this study. However, impacts in the Gulf of Mexico appear to have affected a smaller number of species and species groups, who also contribute greater proportions to biomass, landings, and revenue in this region (e.g., Brown shrimp, Gulf Menhaden). Some post-event increases in values for these species also occurred following these more localized events of shorter duration, and with differential impacts to functional groups. The differing natures and scales of these events may partially explain why these mixed responses were observed in this region compared to others affecting by heatwaves and warming activities. These mixed longer-term effects also may be due to greater species diversity in subtropical systems, which may result in greater stability against press perturbations at some levels [58]. Studies also have observed that increased spatial mobility of harvesters and greater fishery diversification may also enhance resilience to environmental perturbations [74]. However, a number of studies observed short-term effects in biomass, landings, and revenue from both Hurricane Katrina and DWH on Gulf of Mexico fisheries, particularly those for shrimp, blue crab, menhaden, and oyster [89–93]. Furthermore, rebounds to pre-spill levels were observed for a number of taxa, including small pelagic fishes to longer-lived reef fishes, despite significant short-term changes in community structure, while declines for small demersal species also were found to persist in certain reef environments [89–94]. These short-term trends for revenue also were observed in several *post-hoc* investigations [89, 92, 93], as found in this study. Fishing closures and other management actions taken both during and post-spill likely affected biomass, landings, and revenue as well [95–97]. Additionally, impacts from the COVID-19 pandemic in more recent years also may have affected landings and revenue in these systems [97]. Collectively these results are consistent with pelagic or lower trophic level taxa being more susceptible to but also more able to recover from events, and with greater resilience generally observed at the system level.

Being able to detect changes at higher hierarchical levels is quite valuable for many reasons, chief of which is identifying a systemic response much faster than conducting a *post-hoc* meta-analysis of the component taxa [59, 98, 99]. Primarily, these significant responses were observed between pre- and post-event periods at mid-to-lower hierarchical levels, illustrating that these extreme events can alter particular components of the ecosystem despite system-level stability. This work builds upon past aggregative approaches and examined the pelagic to demersal ratio and the percentage of shellfish as key response indicators [59, 98–101], for which post-event significant effects were observed in this study for multiple systems. These variables are indicative of how energy flows through an ecosystem for specific species groups (i.e., as related to life history and habitat), and coupled with the total biomass, landings, or revenue, can provide a relatively rapid way to ascertain what taxa, energy flows, and structural aspects of a food web are dominating an ecosystem. They can also help interpret differential responses when compared across ecosystems. We return to this thesis regarding hierarchical and differential responses below.

Some ecosystems generally exhibited a return to pre-event conditions, implying resilience to these extreme events [33, 71, 85, 102–104]. This pattern was detected by contrasting the pre- and post- event conditions, especially in the higher biological hierarchical levels. This trend occurred across all three of the main response variables, but especially for biomass. The return to pre-event conditions was most prominent in the Gulf of Alaska, followed by the Gulf of Mexico and Gulf of Maine. Inferring that these socio-ecological systems have some degree of resilience is a positive observation for the stability and persistence of key components of marine ecosystems generally, particularly as they are projected to experience increased frequencies of extreme events [6, 8–10, 33, 75, 85, 102, 103]. Being able to identify when an entire ecosystem has recovered from a perturbation is not a trivial result and has high utility in many applications [15, 33, 87–89, 104]. Although management actions or other forcing factors beyond these events could affect ecosystem dynamics, and alter their compositions, these patterns suggest some inherent system-level stability for fisheries systems. Furthermore, resilience at the system level may not necessarily transfer to particular species or groups of species with ecological or economic importance, such as Alaskan snow crab or Pacific cod, whose biomass and landings remain at lower levels post-heatwave [68, 69, 82]. Additionally, factors from climate change, species physiology, fishing practices, ecological interactions, among other considerations within a given system may influence the level of resilience [79, 82, 105]. But this level of systemic or higher aggregate group level stability implies opportunities for fisheries diversification. Conversely, when even higher hierarchical levels or even the total system values of biomass, landings or revenue have significantly shifted, that is a clear signal of major perturbations having occurred that need to be either addressed much more systematically than on a taxa-by-taxa basis or recognized as a shift to new conditions.

Some ecosystems exhibited signs of such a shift to new conditions. Perhaps this implies that a regime shift or change to an alternate steady state has occurred in some of these ecosystems [18–20], an observation that has been documented previously for some of the west coast systems, including disparate responses in kelp beds to marine heatwaves or differential levels of return to pre-DWH spill conditions [83, 89, 94, 95]. This type of pattern also was seen in all three response variables, especially revenue. Temporary fishing closures enacted during the DWH event also may have affected post-event responses in that system [95, 96]. The most prominent shift in ecosystem response was observed in California. Most noteworthy, particularly at the entire system-level, is that a loss of total fisheries landings is not a minor result. This level of total landings may have decreased because of changes in management (e.g., decreased catch limits or fisheries closures) or socioeconomic factors (e.g., shifts in fishing community activity) [33]. However, the responses we observed imply that in some way energy and production have either left the system or are not returning to prior levels [33, 106, 107]. Either way, that is not an insignificant (and mostly likely not a positive) observation for ecosystems that exhibit this type of response to extreme events.

The question begs, why did some ecosystems exhibit resilient responses and others demonstrate signs of a regime shift? Examining the pelagic to demersal ratio, percent shellfish, and specific species may elucidate hypotheses why this could have occurred. In ecosystems dominated by small pelagic species, such as off California, the entire fish community biomass (and associated landings and revenue) may be more susceptible to shorter-term impacts, less resilient to perturbations that last more than a year, and less able to resist system-wide impacts. These factors also may explain the greater magnitude of significant decreases in Gulf of Alaska pelagic biomass post-heatwave. Or it may be that many of the taxa simply migrate to more favorable conditions [33, 58–62]. These responses are due to life history, behavioral, and environmental tolerances well documented as important considerations for small pelagics [33, 62, 65]. These responses would be seen more in biomass and landings, which is what we observed in California. Observed northward migrations of certain small pelagic species from California to the PNW (e.g., California market squid) may further explain the higher level of stability seen in that ecosystem [33].

In ecosystems with a high percentage of shellfish, we would expect to see impacts similar to those for small pelagics on these dominant taxa, largely due to their lower ability to migrate (at least benthic species) and also related to shorter life histories for certain species (e.g., some bivalves, most decapods) [84–86, 108]. Shellfish could be hypothesized to rebound faster under certain conditions, as observed in percentages of crustacean biomass and bivalve landings for the Gulf of Maine, but the short-term responses to event impacts might be even more pronounced as observed for post-event Gulf of Alaska and Gulf of Mexico shellfish landings declines. Given the higher value of these taxa, this would be particularly prevalent in fisheries revenues, which is what we observed. Further, since in some ecosystems the shellfish include a large amount of squids, the response could be amplified by the small pelagic factors noted above. Conversely, in ecosystems dominated by more demersal taxa or that have a lower percentage of shellfish, from the same rationale (especially relatively longer life histories), one would expect more stable responses to perturbations from extreme events [63, 84], which may translate to increased revenue for some of these more resilient species as observed in our study for some regions. The greater integration of multiple year-classes over a longer period of time would give these fisheries systems a greater ability to withstand extreme events, despite potential impacts to varying trophic levels [69, 84, 88].

Several of these ecosystems have experienced notable changes in the past, to the point that some of them are an archetype of regime shifts [109–115]. For example, there have been clear regime shifts in the eastern Bering Sea and Gulf of Alaska [109, 111–113], and well documented shifts from sardines to anchovy in the California Current ecosystem [114, 115]. The Gulf of Maine also has seen significant shifts in demersal or pelagic orientation of its biomass and associated fisheries [79, 116–118], and its finfish and shellfish composition [79, 80], as has the Gulf of Mexico and other systems [119–121]. The salient point being that all of these ecosystems have experienced and are experiencing dynamic situations, some of which have been of the nature of these acute, extreme events. These systems also are projected to experience even more of these events in the future, with contemporary studies suggesting that the Gulf of Alaska and eastern Bering Sea are potentially undergoing a prolonged post-“Blob” status and new heat-related event with subsequent impacts on their fishery ecosystems [67, 71, 72, 85, 122, 123]. Our thesis is that tracking not only taxa of interest, but increasing levels of the biological hierarchy in these systems may provide insights and guidance as to what to expect in these future events.

Ecological theory notes that pulse versus press events, also known as acute versus chronic events, can result in different types of responses [18–20, 60]. As extreme events continue to become more frequent, and perhaps less extreme and hence more chronic, it is highly probable that marine ecosystems will shift from pulse responses to entirely new ecosystem states that persist well beyond any initial conditions and that exhibit less stability and continued, ongoing shifts [18–20, 60, 124]. That is, if extreme events persist as is predicted [6, 125–127], we can reasonably expect an erosion of resilience for these socio-ecological systems [128]. We would then predict that ecosystems dominated by small pelagics, more vulnerable shellfish species, or both could be more susceptible to change into alternate steady states [129, 130]. This response would be seen ultimately in total ecosystem biomass, with the entire system going from being relatively stable due to primary production “table-setting” that sets the levels of biomass, landings and revenue in an ecosystem [35–38], to a less productive and more easily perturbed ecosystem, as influenced by dynamics in primary and higher-level production associated with these responses.

The frequency and intensity of certain extreme events is projected to increase as global change continues [3–7, 125–127]. Our work here demonstrates that the impacts of these extreme events are likely to vary across a wide range of responses. These responses, whether shifting to new conditions or exhibiting resilience will depend upon the composition of the taxa in the ecosystem. The response also will vary depending upon what level one examines the biological hierarchy. We recommend an evaluation across multiple levels of the biological hierarchy in an ecosystem to truly ascertain the degree of resilience and recovery, or the degree of erosion and shift to an alternate state. The relative stability of total ecosystem features affords some hope that overall ecosystems can generally retain certain levels of productivity in light of extreme perturbations. But when we see even total biomass for an entire ecosystem begin to decline, larger concerns emerge that merit priority attention [16, 19, 20, 22, 25, 33, 75, 83, 106, 126, 128, 131].

## Supporting information

S1 Fig Box-whisker plots for biomass, landings, and revenue values per examined fisheries ecosystem over distinct time periods. For each ecosystem (i.e., (a) eastern Bering Sea, Gulf of Alaska, northern California, Pacific Northwest; (b) Gulf of Maine, northern Gulf of Mexico), box-whisker plots are shown for total biomass, landings, and revenue among pre-event, event, and post-event periods. Significant differences among distinct periods per individual analysis of variance test, and post-hoc Tukey tests, are shown with capital letters. (a) For eastern Bering Sea, Gulf of Alaska, northern California, and Pacific Northwest ecosystem, values are shown for time periods ten years prior to the Pacific marine heatwave (“Blob”), over the duration of the heatwave, and post-heatwave. (b) For the Gulf of Maine, values are depicted among time periods ten years prior to the onset of an accelerated warming period for the Gulf of Maine, during the accelerated warming period and prior to a subsequent marine heatwave and noted spike in temperatures, and for years following the heatwave and during the temperature spike. For the northern Gulf of Mexico, values are shown for time periods ten years prior to Hurricane Katrina, during the post-hurricane period prior to the Deepwater Horizon (DWH) oil spill, and post-DWH event.
(DOCX)

S2 Fig Box-whisker plots for pelagic and demersal biomass, landings, and revenue values per examined Pacific fisheries ecosystem over distinct time periods. For each ecosystem (i.e., (a) eastern Bering Sea, Gulf of Alaska, northern California, Pacific Northwest; (b) Gulf of Maine, northern Gulf of Mexico), box-whisker plots are shown for total pelagic and demersal biomass, landings, and revenue among pre-event, event, and post-event periods. Significant differences among distinct periods per individual analysis of variance test, and post-hoc Tukey tests, for pelagic and demersal values are shown with capital letters. (a) For eastern Bering Sea, Gulf of Alaska, northern California, and Pacific Northwest ecosystem, values are shown for time periods ten years prior to the Pacific marine heatwave (“Blob”), over the duration of the heatwave, and post-heatwave. (b) For the Gulf of Maine, values are depicted among time periods ten years prior to the onset of an accelerated warming period for the Gulf of Maine, during the accelerated warming period and prior to a subsequent marine heatwave and noted spike in temperatures, and for years following the heatwave and during the temperature spike. For the northern Gulf of Mexico, values are shown for time periods ten years prior to Hurricane Katrina, during the post-hurricane period prior to the Deepwater Horizon (DWH) oil spill, and post-DWH event.
(DOCX)

S3 Fig Box-whisker plots for shellfish and non-shellfish biomass, landings, and revenue values per examined Pacific fisheries ecosystem over distinct time periods. For each ecosystem (i.e., (a) eastern Bering Sea, Gulf of Alaska, northern California, Pacific Northwest; (b) Gulf of Maine, northern Gulf of Mexico), box-whisker plots are shown for total shellfish (i.e., crustaceans and bivalves) and non-shellfish biomass, landings, and revenue among pre-event, event, and post-event periods. Significant differences among distinct periods per individual analysis of variance test, and post-hoc Tukey tests, for shellfish and non-shellfish values are shown with capital letters. (a) For eastern Bering Sea, Gulf of Alaska, northern California, and Pacific Northwest ecosystem, values are shown for time periods ten years prior to the Pacific marine heatwave (“Blob”), over the duration of the heatwave, and post-heatwave. (b) For the Gulf of Maine, values are depicted among time periods ten years prior to the onset of an accelerated warming period for the Gulf of Maine, during the accelerated warming period and prior to a subsequent marine heatwave and noted spike in temperatures, and for years following the heatwave and during the temperature spike. For the northern Gulf of Mexico, values are shown for time periods ten years prior to Hurricane Katrina, during the post-hurricane period prior to the Deepwater Horizon (DWH) oil spill, and post-DWH event.
(DOCX)

S4 Fig Trends for major families in response to (a) the Pacific marine heatwave (“Blob”) during distinct time periods and (b) the Gulf of Maine regional warming and Gulf of Mexico extreme events during distinct time periods. Average biomass, landings, and revenue values are shown for major families (i.e., top contributors to total biomass, landings, and revenue) during distinct time periods. (a) For eastern Bering Sea, Gulf of Alaska, northern California, and Pacific Northwest ecosystems, values are shown during distinct time periods ten years prior to the Pacific marine heatwave (“Blob”), over the duration of the heatwave, and post-heatwave. (b) For the Gulf of Maine ecosystem, values are depicted for time periods ten years prior to the onset of an accelerated warming period for the Gulf of Maine, during the accelerated warming period and prior to a subsequent marine heatwave and noted spike in temperatures, and for years following the heatwave and during the temperature spike. For the northern Gulf of Mexico, values are shown for time periods ten years prior to Hurricane Katrina, during the post-hurricane period prior to the Deepwater Horizon (DWH) oil spill, and for years following the DWH event.
(DOCX)

S1 Table Tests of independence between Sea Around Us annual fisheries landings data (used as a proxy for biomass in this study) and National Oceanic and Atmospheric Administration (NOAA) annual fisheries landings data. For each region (i.e., Alaska, northern California, Pacific Northwest, Gulf of Maine, northern Gulf of Mexico), t and p-values (t-test), D and p-value (Komolgorov-Smirnov test; K-S test), and Pearson correlation coefficients (Coeff) and p-values (time series cross-comparisons) are provided for each analysis. For Alaska, data were compared at the statewide scale due to reporting of NOAA landings at the statewide level.
(DOCX)

S2 Table Multivariate analysis of variance results for total biomass, landings, and revenue values per examined fisheries ecosystem over time. For each ecosystem (i.e., eastern Bering Sea, Gulf of Alaska, northern California, Pacific Northwest, Gulf of Maine, northern Gulf of Mexico), F and p-values are provided for each multivariate analysis of variance model to examine significant differences in total biomass, landings, and revenue among periods (i.e., pre-event, event, and post-event), associated with all three variables concurrently over time. Bold values indicate statistically significant relationships (*p≤0.05; **p≤0.01; ***p≤0.001).
(DOCX)

S3 Table Multivariate analysis of variance results for pelagic to demersal ratios and percent shellfish of biomass, landings, and revenue values per examined fisheries ecosystem over time. For each ecosystem (i.e., eastern Bering Sea, Gulf of Alaska, northern California, Pacific Northwest, Gulf of Maine, northern Gulf of Mexico), F and p-values are provided for each multivariate analysis of variance model to examine significant differences in pelagic to demersal ratios and percent shellfish of biomass, landings, and revenue among periods (i.e., pre-event, event, and post-event), associated with all three variables concurrently over time. Bold values indicate statistically significant relationships (*p≤0.05; **p≤0.01; ***p≤0.001).
(DOCX)

S4 Table Global R and global p-values for analysis of similarity tests. Global R and global p-values (in parenthesis) for all analysis of similarity tests to examine significant differences in species composition for biomass, landings, and revenue among pre-event, event, and post-event periods. For eastern Bering Sea, Gulf of Alaska, northern California, and Pacific Northwest ecosystems, tests of significance in values were conducted for time periods ten years prior to the Pacific marine heatwave (“Blob”), over the duration of the heatwave, and post-heatwave. For the Gulf of Maine, differences in values were examined among time periods ten years prior to the onset of an accelerated warming period for the Gulf of Maine, during the accelerated warming period and prior to a subsequent marine heatwave and noted spike in temperatures, and for years following the heatwave and during the temperature spike. For the northern Gulf of Mexico, tests were conducted for time periods ten years prior to Hurricane Katrina, during the post-hurricane period prior to the Deepwater Horizon (DWH) oil spill, and post-DWH event. Bold values indicate statistically significant relationships.
(DOCX)

S5 Table By region, similarity percentages (SIMPER) results tables for all significant relationships observed among examined time periods. Average dissimilarity (Av. Dissim), percent contribution (Contrib. %), and cumulative percent contribution (Cumul. %) for all major species (i.e., cumulatively up to 90% through SIMPER analysis) contributing to significant differences in species composition for biomass, landings, and revenue among pre-event, event, and post-event periods (as examined through analysis of similarity testing). For eastern Bering Sea (EBS), Gulf of Alaska (GAK), northern California, and Pacific Northwest ecosystems, tests of significance in values were conducted for time periods ten years prior to the Pacific marine heatwave (“Blob”), over the duration of the heatwave, and post-heatwave. For the Gulf of Maine, differences in values were examined among time periods ten years prior to the onset of an accelerated warming period for the Gulf of Maine, during the accelerated warming period and prior to a subsequent marine heatwave and noted spike in temperatures, and for years following the heatwave and during the temperature spike. For the northern Gulf of Mexico, tests were conducted for time periods ten years prior to Hurricane Katrina, during the post-hurricane period prior to the Deepwater Horizon (DWH) oil spill, and post-DWH event. All species with Contrib. % values greater than 1.0 are in bold. Italicized species names indicate that a mean increase (shown as +) was observed between at least one period for a species with a Contib. % greater than 1.0 for a given variable.
(DOCX)
