# Supplementary material for: Responses of fisheries ecosystems to marine heatwaves and other extreme events
Source: PLoS One. 2024 Dec 6;19(12):e0315224. doi: 10.1371/journal.pone.0315224 (PMC11623807; doi:10.1371/journal.pone.0315224)
Supplement: S3 Fig — For each ecosystem (i.e., (a) eastern Bering Sea, Gulf of Alaska, northern California, Pacific Northwest; (b) Gulf of Maine, northern Gulf of Mexico), box-whisker plots are shown for total shellfish (i.e., crustaceans and bivalves) and non-shellfish biomass, landings, and revenue among pre-event, event, and post-event periods. Significant differences among distinct periods per individual analysis of variance test, and post-hoc Tukey tests, for shellfish and non-shellfish values are shown with capital letters. (a) For eastern Bering Sea, Gulf of Alaska, northern California, and Pacific Northwest ecosystem, values are shown for time periods ten years prior to the Pacific marine heatwave (“Blob”), over the duration of the heatwave, and post-heatwave. (b) For the Gulf of Maine, values are depicted among time periods ten years prior to the onset of an accelerated warming period for the Gulf of Maine, during the accelerated warming period and prior to a subsequent marine heatwave and noted spike in temperatures, and for years following the heatwave and during the temperature spike. For the northern Gulf of Mexico, values are shown for time periods ten years prior to Hurricane Katrina, during the post-hurricane period prior to the Deepwater Horizon (DWH) oil spill, and post-DWH event. (DOCX) [file pone.0315224.s003.docx]

Supplementary Materials for

**Responses of fisheries ecosystems to marine heatwaves and other extreme events**

Anthony R. Marshak, Jason S. Link

*Corresponding author. Email: [tmarshak62@gmail.com](mailto:tmarshak62@gmail.com)

**This PDF file includes:**

S3 Fig.


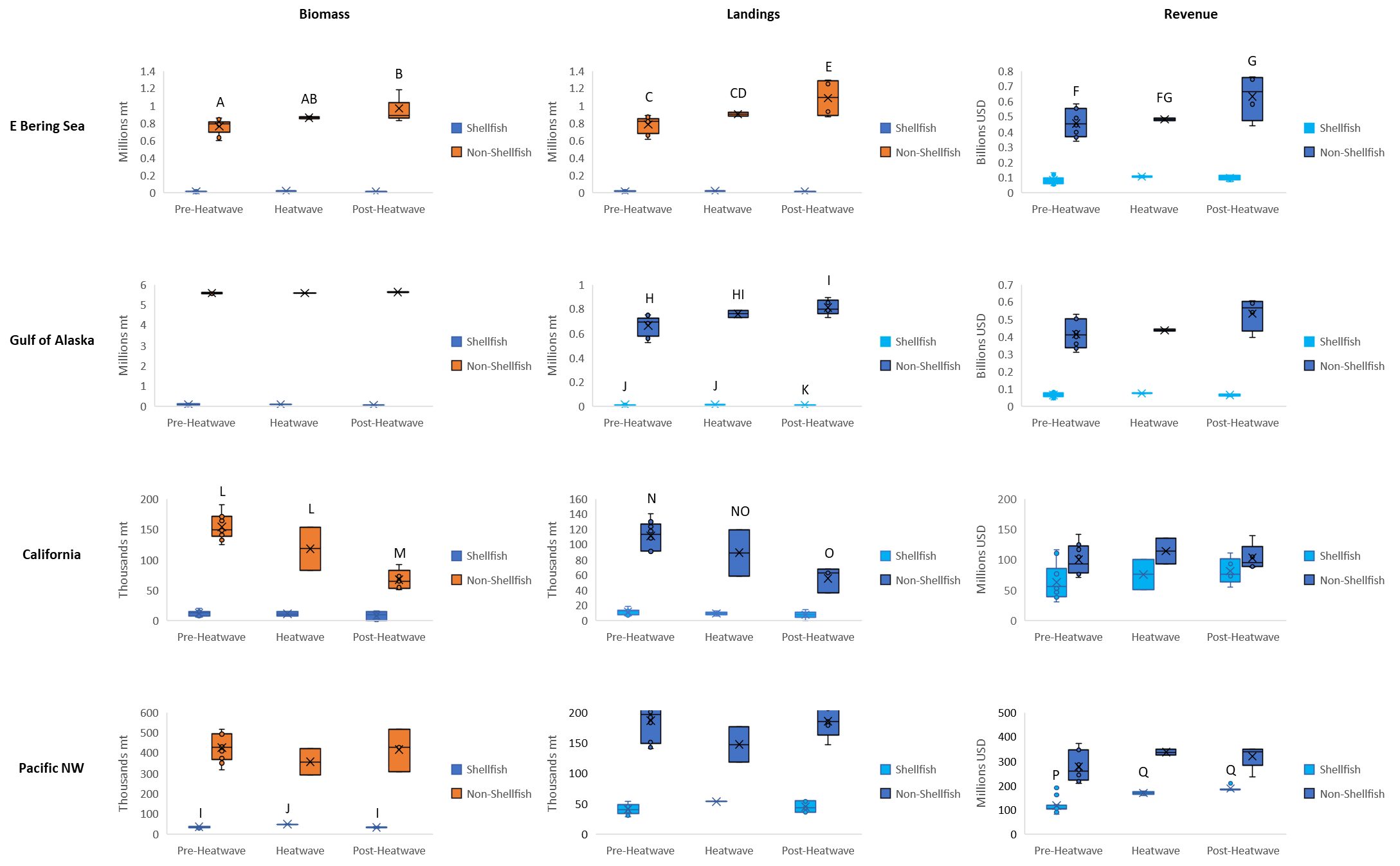


S3a Fig. Box-Whisker Plots for shellfish and non-shellfish biomass, landings, and revenue values per examined Pacific fisheries ecosystem over distinct time periods. For each ecosystem (i.e., eastern Bering Sea, Gulf of Alaska, northern California, Pacific Northwest), box-whisker plots are shown for total shellfish (i.e., crustaceans and bivalves) and non-shellfish biomass, landings, and revenue among pre-event, event, and post-event periods. Significant differences among distinct periods per individual analysis of variance test, and post-hoc Tukey tests, for shellfish and non-shellfish values are shown with capital letters. For eastern Bering Sea, Gulf of Alaska, northern California, and Pacific Northwest ecosystem, values are shown for time periods ten years prior to the Pacific marine heatwave (“Blob”), over the duration of the heatwave, and post-heatwave.


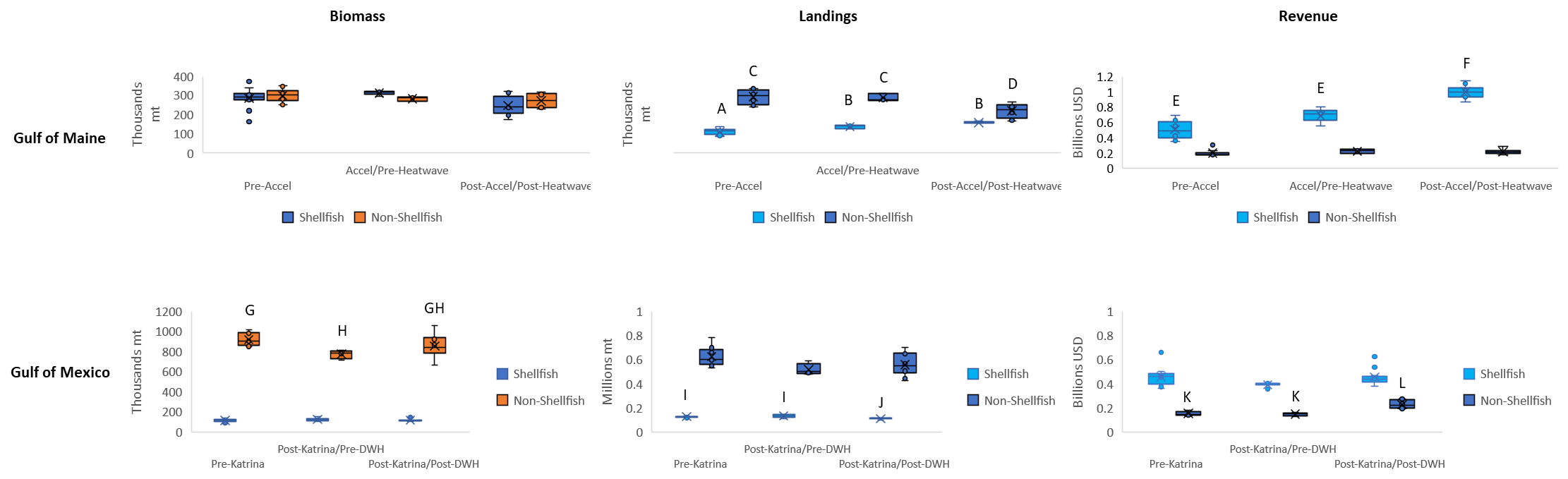


S3b Fig. Box-Whisker Plots for shellfish and non-shellfish biomass, landings, and revenue values per examined Atlantic fisheries ecosystem over distinct time periods. For each ecosystem (i.e., Gulf of Maine, northern Gulf of Mexico), box-whisker plots are shown for total shellfish (i.e., crustaceans and bivalves) and non-shellfish biomass, landings, and revenue among pre-event, event, and post-event periods. Significant differences among distinct periods per individual analysis of variance test, and post-hoc Tukey tests, for shellfish and non-shellfish values are shown with capital letters. For the Gulf of Maine, values are depicted among time periods ten years prior to the onset of an accelerated warming period for the Gulf of Maine, during the accelerated warming period and prior to a subsequent marine heatwave and noted spike in temperatures, and for years following the heatwave and during the temperature spike. For the northern Gulf of Mexico, values are shown for time periods ten years prior to Hurricane Katrina, during the post-hurricane period prior to the Deepwater Horizon (DWH) oil spill, and post-DWH event.
