# Supplementary material for: Responses of fisheries ecosystems to marine heatwaves and other extreme events
Source: PLoS One. 2024 Dec 6;19(12):e0315224. doi: 10.1371/journal.pone.0315224 (PMC11623807; doi:10.1371/journal.pone.0315224)
Supplement: S4 Fig — Average biomass, landings, and revenue values are shown for major families (i.e., top contributors to total biomass, landings, and revenue) during distinct time periods. (a) For eastern Bering Sea, Gulf of Alaska, northern California, and Pacific Northwest ecosystems, values are shown during distinct time periods ten years prior to the Pacific marine heatwave (“Blob”), over the duration of the heatwave, and post-heatwave. (b) For the Gulf of Maine ecosystem, values are depicted for time periods ten years prior to the onset of an accelerated warming period for the Gulf of Maine, during the accelerated warming period and prior to a subsequent marine heatwave and noted spike in temperatures, and for years following the heatwave and during the temperature spike. For the northern Gulf of Mexico, values are shown for time periods ten years prior to Hurricane Katrina, during the post-hurricane period prior to the Deepwater Horizon (DWH) oil spill, and for years following the DWH event. (DOCX) [file pone.0315224.s004.docx]

Supplementary Materials for

**Responses of fisheries ecosystems to marine heatwaves and other extreme events**

Anthony R. Marshak, Jason S. Link

*Corresponding author. Email: [tmarshak62@gmail.com](mailto:tmarshak62@gmail.com)

**This PDF file includes:**

S4 Fig.


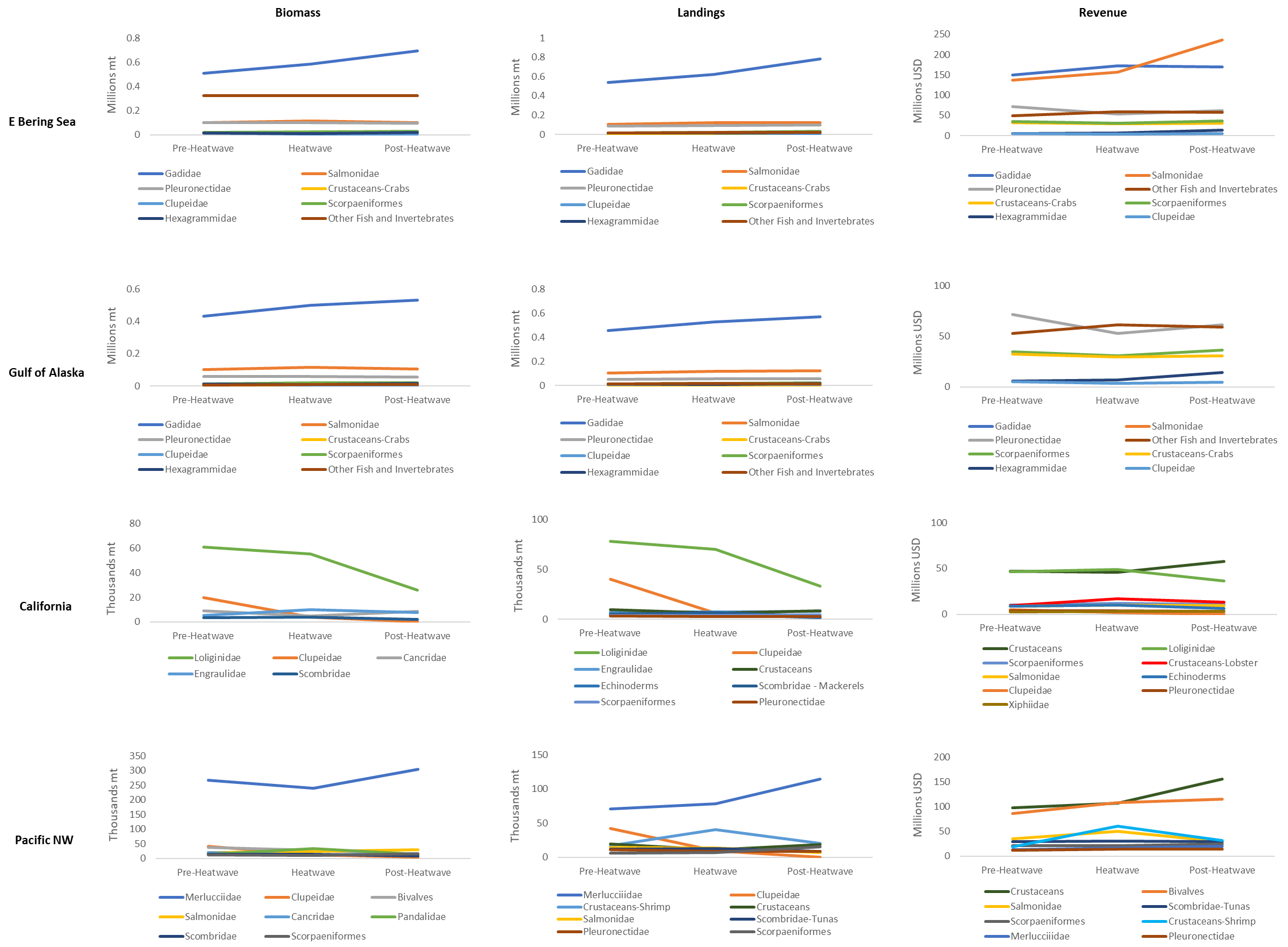


S4a Fig. Trends for major families in response to the Pacific marine heatwave (“Blob”) during distinct time periods. Average biomass, landings, and revenue values are shown for major families (i.e., top contributors to total biomass, landings, and revenue) distinct time periods. For eastern Bering Sea, Gulf of Alaska, northern California, and Pacific Northwest ecosystems, values are shown for major families (i.e., top contributors to total biomass, landings, and revenue) during distinct time periods ten years prior to the Pacific marine heatwave (“Blob”), over the duration of the heatwave, and post-heatwave).


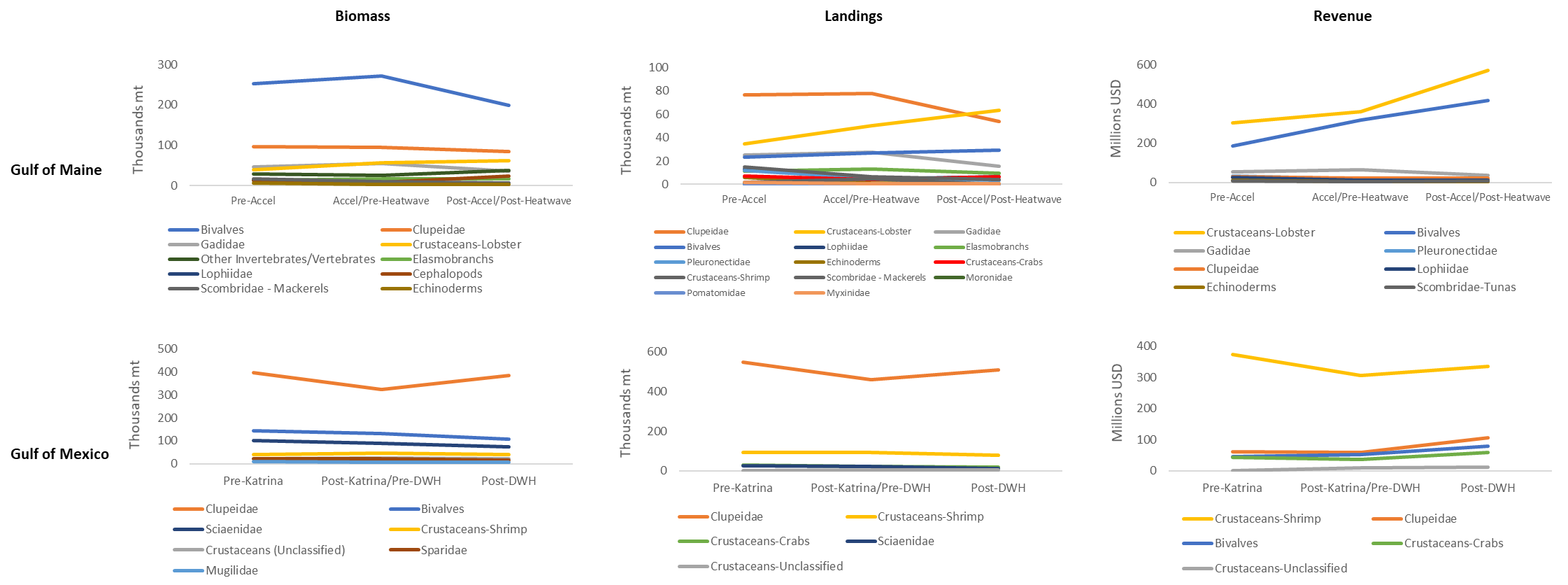


S4b Fig. Trends for major families in response to the Gulf of Maine regional warming and Gulf of Mexico extreme events during distinct time periods. Average biomass, landings, and revenue values are shown for major families (i.e., top contributors to total biomass, landings, and revenue) during distinct time periods. For the Gulf of Maine ecosystem, values are depicted for time periods ten years prior to the onset of an accelerated warming period for the Gulf of Maine, during the accelerated warming period and prior to a subsequent marine heatwave and noted spike in temperatures, and for years following the heatwave and during the temperature spike. For the northern Gulf of Mexico, values are shown for time periods ten years prior to Hurricane Katrina, during the post-hurricane period prior to the Deepwater Horizon (DWH) oil spill, and for years following the DWH event.
