# Supplementary material for: Responses of fisheries ecosystems to marine heatwaves and other extreme events
Source: PLoS One. 2024 Dec 6;19(12):e0315224. doi: 10.1371/journal.pone.0315224 (PMC11623807; doi:10.1371/journal.pone.0315224)
Supplement: S1 Table — For each region (i.e., Alaska, northern California, Pacific Northwest, Gulf of Maine, northern Gulf of Mexico), t and p-values (t-test), D and p-value (Komolgorov-Smirnov test; K-S test), and Pearson correlation coefficients (Coeff) and p-values (time series cross-comparisons) are provided for each analysis. For Alaska, data were compared at the statewide scale due to reporting of NOAA landings at the statewide level. (DOCX) [file pone.0315224.s005.docx]

Supplementary Materials for

**Responses of fisheries ecosystems to marine heatwaves and other extreme events**

Anthony R. Marshak, Jason S. Link

*Corresponding author. Email: [tmarshak62@gmail.com](mailto:tmarshak62@gmail.com)

**This PDF file includes:**

S1 Table.

**S1 Table. Tests of Independence between Sea Around Us annual fisheries landings data (used as a proxy for biomass in this study) and National Oceanic and Atmospheric Administration (NOAA) annual fisheries landings data.**

|  | **t-test** | | **K-S Test** | | **Cross Comparison** | |
| --- | --- | --- | --- | --- | --- | --- |
| **Region** | **t** | **P** | **D** | **P** | **Coeff** | **P** |
| Alaska | 5.674 | <0.0001 | 0.467 | <0.0001 | 0.4998 | <0.0001 |
| California | 6.862 | <0.0001 | 0.52 | <0.0001 | 0.854 | <0.0001 |
| Pacific Northwest | 14.246 | <0.0001 | 0.769 | <0.0001 | 0.449 | <0.0001 |
| Gulf of Maine | 20.708 | <0.0001 | 1 | <0.0001 | -0.214 | 0.077 |
| Gulf of Mexico | 10.542 | <0.0001 | 0.729 | <0.0001 | 0.833 | <0.0001 |

For each region (i.e., Alaska, northern California, Pacific Northwest, Gulf of Maine, northern Gulf of Mexico), t and p-values (t-test), D and p-

value (Komolgorov-Smirnov test; K-S test), and Pearson correlation coefficients (Coeff) and p-values (time series cross-comparisons) are

provided for each analysis. For Alaska, data were compared at the statewide scale due to reporting of NOAA landings at the statewide

level.
