# Supplementary material for: Responses of fisheries ecosystems to marine heatwaves and other extreme events
Source: PLoS One. 2024 Dec 6;19(12):e0315224. doi: 10.1371/journal.pone.0315224 (PMC11623807; doi:10.1371/journal.pone.0315224)
Supplement: S2 Table — For each ecosystem (i.e., eastern Bering Sea, Gulf of Alaska, northern California, Pacific Northwest, Gulf of Maine, northern Gulf of Mexico), F and p-values are provided for each multivariate analysis of variance model to examine significant differences in total biomass, landings, and revenue among periods (i.e., pre-event, event, and post-event), associated with all three variables concurrently over time. Bold values indicate statistically significant relationships (*p≤0.05; **p≤0.01; ***p≤0.001). (DOCX) [file pone.0315224.s006.docx]

Supplementary Materials for

**Responses of fisheries ecosystems to marine heatwaves and other extreme events**

Anthony R. Marshak, Jason S. Link

*Corresponding author. Email: [tmarshak62@gmail.com](mailto:tmarshak62@gmail.com)

**This PDF file includes:**

S2 Table.

**S2 Table.** **Multivariate analysis of variance results for total biomass, landings, and revenue values per examined fisheries ecosystem over time.**

| **Region** | **Wilks' Lambda** | **F** | **P** |
| --- | --- | --- | --- |
| E Bering Sea | 0.4755 | 1.651 | 0.1806 |
| Gulf of Alaska | 0.5237 | 1.4 | 0.2589 |
| California | 0.1326 | 6.984 | **0.0002***** |
| Gulf of Maine | 0.1647 | 7.807 | **<0.0001***** |
| Gulf of Mexico | 0.5159 | 3.922 | **0.0089**** |
| Pacific NW | 0.3218 | 3.051 | **0.0231*** |

For each ecosystem (i.e., eastern Bering Sea, Gulf of Alaska, northern California, Pacific Northwest, Gulf of Maine, northern Gulf of Mexico), F and p-

values are provided for each multivariate analysis of variance model to examine significant differences in total biomass, landings, and revenue among periods

(i.e., pre-event, event, and post-event), associated with all three variables concurrently over time. Bold values indicate statistically significant relationships

(*p≤0.05; **p≤0.01; ***p≤0.001).
