# Supplementary material for: Responses of fisheries ecosystems to marine heatwaves and other extreme events
Source: PLoS One. 2024 Dec 6;19(12):e0315224. doi: 10.1371/journal.pone.0315224 (PMC11623807; doi:10.1371/journal.pone.0315224)
Supplement: S3 Table — For each ecosystem (i.e., eastern Bering Sea, Gulf of Alaska, northern California, Pacific Northwest, Gulf of Maine, northern Gulf of Mexico), F and p-values are provided for each multivariate analysis of variance model to examine significant differences in pelagic to demersal ratios and percent shellfish of biomass, landings, and revenue among periods (i.e., pre-event, event, and post-event), associated with all three variables concurrently over time. Bold values indicate statistically significant relationships (*p≤0.05; **p≤0.01; ***p≤0.001). (DOCX) [file pone.0315224.s007.docx]

Supplementary Materials for

**Responses of fisheries ecosystems to marine heatwaves and other extreme events**

Anthony R. Marshak, Jason S. Link

*Corresponding author. Email: [tmarshak62@gmail.com](mailto:tmarshak62@gmail.com)

**This PDF file includes:**

S3 Table.

S3a Table. Multivariate analysis of variance results for pelagic to demersal ratios and percent shellfish of biomass, landings, and revenue values per examined fisheries ecosystem over time.

|  | **Pelagic/Demersal Ratios** | | | |  | **Percent Shellfish** | | | |
| --- | --- | --- | --- | --- | --- | --- | --- | --- | --- |
| **Region** | | **Wilks’ Lambda** | **F** | **P** | | | **Wilks’ Lambda** | **F** | **P** |
| E Bering Sea | | 0.4263 | 1.949 | 0.1174 | | | 0.3421 | 2.602 | **0.0464*** |
| Gulf of Alaska | | 0.4408 | 1.856 | 0.1343 | | | 0.3908 | 2.199 | 0.0820 |
| California | | 0.2113 | 4.701 | **0.0027**** | | | 0.3016 | 3.284 | **0.0168*** |
| Gulf of Maine | | 0.6714 | 1.176 | 0.3437 | | | 0.1122 | 10.59 | **<0.0001***** |
| Gulf of Mexico | | 0.5289 | 2.375 | **0.0479*** | | | 0.4215 | 3.422 | **0.0084**** |
| Pacific NW | | 0.4308 | 2.094 | 0.0917 | | | 0.1425 | 6.595 | **0.0003***** |

For each ecosystem (i.e., eastern Bering Sea, Gulf of Alaska, northern California, Pacific Northwest, Gulf of Maine, northern Gulf of Mexico), F and p-

values are provided for each multivariate analysis of variance model to examine significant differences in pelagic to demersal ratios and percent shellfish of

biomass, landings, and revenue among periods (i.e., pre-event, event, and post-event), associated with all three variables concurrently over time. Bold values

indicate statistically significant relationships (*p≤0.05; **p≤0.01; ***p≤0.001).

S3b Table. Analysis of variance results for pelagic to demersal ratios and percent shellfish of biomass, landings, and revenue values per examined fisheries ecosystem over time.

| **Region** | **Biomass** |  | **Landings** |  | **Revenue** |  |
| --- | --- | --- | --- | --- | --- | --- |
| *Pelagic/Demersal Ratio* | **F** | **P** | **F** | **P** | **F** | **P** |
| E Bering Sea | 8.766 | **0.0045**** | 2.826 | 0.0957 | 2.666 | 0.1071 |
| Gulf of Alaska | 7.395 | **0.0081**** | 2.067 | 0.1661 | 2.021 | 0.1721 |
| California | 13.5 | **0.0005***** | 1.553 | 0.2546 | 3.545 | 0.0568 |
| Gulf of Maine | 2.889 | 0.0849 | 4.119 | **0.0337*** | 1.441 | 0.2628 |
| Gulf of Mexico | 6.837 | **0.0058**** | 1.25851 | **0.0060**** | 7.426 | **0.0036**** |
| Pacific NW | 0.9342 | 0.4197 | 0.818 | 0.4613 | 2.787 | 0.0957 |

| *Percent Shellfish* | **F** | **P** | | **F** | | **P** | | **F** | | **P** |
| --- | --- | --- | --- | --- | --- | --- | --- | --- | --- | --- |
| E Bering Sea | 0.2337 | | 0.7951 | | 0.4631 | | 0.6393 | | 0.2043 | 0.8178 |
| Gulf of Alaska | 7.948 | **0.0063**** | | 5.225 | | **0.0216*** | | 0.0995 | | 0.9059 |
| California | 7.352 | **0.0082**** | | 9.668 | | **0.0023**** | | 6.829 | | **0.0085**** |
| Gulf of Maine | 0.4745 | 0.6307 | | 51.62 | | **<0.0001***** | | 21.11 | | **<0.0001***** |
| Gulf of Mexico | 6.398 | **0.0075**** | | 4.225 | | **0.0287*** | | 6.518 | | **0.0062**** |
| Pacific NW | 10.13 | **0.0026**** | | 8.947 | | **0.0031**** | | 14.7 | | **0.0004***** |

For each ecosystem (i.e., eastern Bering Sea, Gulf of Alaska, northern California, Pacific Northwest, Gulf of Maine, northern Gulf of Mexico), F and p-

values are provided for each analysis of variance model to examine significant differences in pelagic to demersal ratios and percent shellfish of biomass,

landings, and revenue among periods (i.e., pre-event, event, and post-event), associated with all three variables concurrently over time. For eastern Bering

Sea, Gulf of Alaska, northern California, and Pacific Northwest regions, tests of significance in values were conducted for time periods ten years prior to the

Pacific marine heatwave (“Blob”), over the duration of the heatwave, and post-heatwave. For the Gulf of Maine, differences in values were examined among

time periods ten years prior to the onset of an accelerated warming period for the Gulf of Maine, during the accelerated warming period and prior to a

subsequent marine heatwave and noted spike in temperatures, and for years following the heatwave and during the temperature spike. For the northern Gulf

of Mexico, tests were conducted for time periods ten years prior to Hurricane Katrina, during the post-hurricane period prior to the Deepwater Horizon

(DWH) oil spill, and post-DWH event. Bold values indicate statistically significant relationships (*p≤0.05; **p≤0.01; ***p≤0.001).
