# Supplementary material for: Responses of fisheries ecosystems to marine heatwaves and other extreme events
Source: PLoS One. 2024 Dec 6;19(12):e0315224. doi: 10.1371/journal.pone.0315224 (PMC11623807; doi:10.1371/journal.pone.0315224)
Supplement: S4 Table — Global R and global p-values (in parenthesis) for all analysis of similarity tests to examine significant differences in species composition for biomass, landings, and revenue among pre-event, event, and post-event periods. For eastern Bering Sea, Gulf of Alaska, northern California, and Pacific Northwest ecosystems, tests of significance in values were conducted for time periods ten years prior to the Pacific marine heatwave (“Blob”), over the duration of the heatwave, and post-heatwave. For the Gulf of Maine, differences in values were examined among time periods ten years prior to the onset of an accelerated warming period for the Gulf of Maine, during the accelerated warming period and prior to a subsequent marine heatwave and noted spike in temperatures, and for years following the heatwave and during the temperature spike. For the northern Gulf of Mexico, tests were conducted for time periods ten years prior to Hurricane Katrina, during the post-hurricane period prior to the Deepwater Horizon (DWH) oil spill, and post-DWH event. Bold values indicate statistically significant relationships. (DOCX) [file pone.0315224.s008.docx]

Supplementary Materials for

**Responses of fisheries ecosystems to marine heatwaves and other extreme events**

Anthony R. Marshak, Jason S. Link

*Corresponding author. Email: [tmarshak62@gmail.com](mailto:tmarshak62@gmail.com)

**This PDF file includes:**

S4 Table.

S4 Table. Global R and global p-values for analysis of similarity tests.

|  | **E Bering Sea** | **Gulf of Alaska** | **California** | **Pacific NW** | **Gulf of Maine** | **Gulf of Mexico** |
| --- | --- | --- | --- | --- | --- | --- |
| Biomass | R=0.1195 (p=0.2582) | R=0.0204 (p=0.3883) | R=0.6634 (**p=0.0002**) | R=0.4832 (**p=0.0172**) | R=0.5705 (**p=0.0003**) | R=0.3573 (**p=0.0004**) |
| Landings | R=0.2178 (p=0.1004) | R=0.0498 (p=0.3141) | R=0.4643 (**p=0.0080**) | R=0.4804 (**p=0.0019**) | R=0.663 (**p=0.0001**) | R=0.1855 (**p=0.0206**) |
| Revenue | R=0.194 (p=0.1118) | R=0.2828 (**p=0.0365**) | R=0.3054 (**p=0.0099**) | R=0.4723 (**p=0.0016**) | R=0.684 (**p=0.0001**) | R=0.6578 (**p=0.0001**) |

Global R and global p-values (in parenthesis) for all analysis of similarity tests to examine significant differences in species composition for biomass,

landings, and revenue among pre-event, event, and post-event periods. For eastern Bering Sea, Gulf of Alaska, northern California, and Pacific Northwest

ecosystems, tests of significance in values were conducted for time periods ten years prior to the Pacific marine heatwave (“Blob”), over the duration of the

heatwave, and post-heatwave. For the Gulf of Maine, differences in values were examined among time periods ten years prior to the onset of an accelerated

warming period for the Gulf of Maine, during the accelerated warming period and prior to a subsequent marine heatwave and noted spike in temperatures,

and for years following the heatwave and during the temperature spike. For the northern Gulf of Mexico, tests were conducted for time periods ten years prior

to Hurricane Katrina, during the post-hurricane period prior to the Deepwater Horizon (DWH) oil spill, and post-DWH event. Bold values indicate

statistically significant relationships.
