# Supplementary material for: Responses of fisheries ecosystems to marine heatwaves and other extreme events
Source: PLoS One. 2024 Dec 6;19(12):e0315224. doi: 10.1371/journal.pone.0315224 (PMC11623807; doi:10.1371/journal.pone.0315224)
Supplement: S5 Table — Average dissimilarity (Av. Dissim), percent contribution (Contrib. %), and cumulative percent contribution (Cumul. %) for all major species (i.e., cumulatively up to 90% through SIMPER analysis) contributing to significant differences in species composition for biomass, landings, and revenue among pre-event, event, and post-event periods (as examined through analysis of similarity testing). For eastern Bering Sea (EBS), Gulf of Alaska (GAK), northern California, and Pacific Northwest ecosystems, tests of significance in values were conducted for time periods ten years prior to the Pacific marine heatwave (“Blob”), over the duration of the heatwave, and post-heatwave. For the Gulf of Maine, differences in values were examined among time periods ten years prior to the onset of an accelerated warming period for the Gulf of Maine, during the accelerated warming period and prior to a subsequent marine heatwave and noted spike in temperatures, and for years following the heatwave and during the temperature spike. For the northern Gulf of Mexico, tests were conducted for time periods ten years prior to Hurricane Katrina, during the post-hurricane period prior to the Deepwater Horizon (DWH) oil spill, and post-DWH event. All species with Contrib. % values greater than 1.0 are in bold. Italicized species names indicate that a mean increase (shown as +) was observed between at least one period for a species with a Contib. % greater than 1.0 for a given variable. (DOCX) [file pone.0315224.s009.docx]

Supplementary Materials for

**Responses of fisheries ecosystems to marine heatwaves and other extreme events**

Anthony R. Marshak, Jason S. Link

*Corresponding author. Email: [tmarshak62@gmail.com](mailto:tmarshak62@gmail.com)

**This PDF file includes:**

S5 Table.

S5 Table. By region, similarity percentages (SIMPER) results tables for all significant relationships observed among examined time periods.

| **Gulf of Alaska^[[1]](#footnote-1)^** | **GAK, EBS Biomass** | **EBS Landings** | | | **GAK Landings** | **EBS Revenue** | | | **GAK Revenue** | | | | | | |  |
| --- | --- | --- | --- | --- | --- | --- | --- | --- | --- | --- | --- | --- | --- | --- | --- | --- |
| **E Bering Sea^[[2]](#footnote-2)^** | **NS** | **Pre-Heat v. Post-Heat** | | | **NS** | **Pre-Heat v. Post-Heat** | | | **Pre-Heat v. Post-Heat** | | | | | | |  |
| **Taxon** |  | **Av. Dissim** | **Contrib. %** | **Cumul. %** |  | **Av. Dissim** | **Contrib. %** | **Cumul. %** | **Av. Dissim** | **Contrib. %** | | | | **Cumul. %** | |  |
| ***Sockeye Salmon*** |  | 0.6922 | **3.545 (+)** | 3.55 |  | 7.717 | **23.96 (+)** | 23.96 | 7.459 | **31.83 (+)** | | | 31.83 | | |  |
| ***Walleye Pollock*** |  | 11.97 | **61.33 (+)** | 64.88 |  | 6.27 | **19.47 (+)** | 43.43 | 2.739 | **11.69 (+)** | | | 43.52 | | |  |
| ***Pink Salmon*** |  | 1.414 | **7.245** | 72.12 |  | 2.111 | **6.555 (+)** | 49.99 | 2.161 | **9.22 (+)** | | | 52.74 | | |  |
| **Pacific Halibut** |  | 0.2463 | **1.261** | 73.38 |  | 2.057 | **6.388** | 56.37 | 1.657 | **7.073** | | | 59.82 | | |  |
| ***Chum Salmon*** |  | 0.3386 | **1.734 (+)** | 75.12 |  | 2.017 | **6.264 (+)** | 62.64 | 1.54 | **6.572 (+)** | | | 66.39 | | |  |
| ***Pacific Cod*** |  | 0.9663 | **4.949 (+)** | 80.06 |  | 1.659 | **5.15 (+)** | 67.79 | 1.269 | **5.415 (+)** | | | 71.8 | | |  |
| **Snow/Tanner Crab** |  | 0.2868 | **1.469** | 81.53 |  | 1.513 | **4.697** | 72.49 | 1.02 | **4.352 (+)** | | | 76.16 | | |  |
| ***King Crab*** |  | 0.04849 | 0.2483 | 81.78 |  | 1.511 | **4.691 (+)** | 77.18 | 0.9156 | **3.908** | | | 80.06 | | |  |
| ***Atka Mackerel*** |  | 0.3758 | **1.925 (+)** | 83.71 |  | 0.9685 | **3.007 (+)** | 80.18 | 0.7803 | **3.33 (+)** | | | 83.39 | | |  |
| ***Dungeness Crab*** |  | - | **-** | **-** |  | **-** | **-** | **-** | 0.5842 | **2.493 (+)** | | | 85.89 | | | |
| ***Pac. Ocean Perch*** |  | 0.7841 | **4.016 (+)** | 87.72 |  | 0.6737 | **2.092 (+)** | 82.27 | 0.3159 | **1.348 (+)** | | | 87.24 | | | |
| ***Sablefish*** |  | 0.04586 | 0.2349 | 87.96 |  | 0.6367 | **1.977 (+)** | 84.25 | 0.571 | | **2.437 (+)** | 89.68 | | | |  |
| ***Yellowfin Sole*** |  | 0.9569 | **4.901 (+)** | 92.86 |  | 1.187 | **3.685 (+)** | 87.94 | 0.546 | **2.33 (+)** | | | | 92.01 |  |  |

| **California^[[3]](#footnote-3)^** | **Biomass** | | | | | | | | | | **Landings** | | |
| --- | --- | --- | --- | --- | --- | --- | --- | --- | --- | --- | --- | --- | --- |
|  | **Pre-Heatwave v. Heatwave** | | | | | | **Pre-Heatwave v. Post-Heatwave** | | | | **Pre-Heatwave v. Post-Heatwave** | | |
| **Taxon** | **Av. Dissim** | | **Contrib. %** | | **Cumul. %** | | **Av. Dissim** | | **Contrib. %** | **Cumul. %** | **Av. Dissim** | **Contrib. %** | **Cumul. %** |
| **California Market Squid** | 13.11 | | **35.08** | | 35.08 | | 19.88 | | **39.50** | 39.50 | 22.82 | **45.00** | 45.00 |
| **Pacific Sardine** | 12.27 | | **32.83** | | 67.91 | | 16.21 | | **32.20** | 71.70 | 11.26 | **22.21** | 67.21 |
| ***Northern Anchovy*** | 2.687 | | **7.19 (+)** | | 75.1 | | 2.298 | | **4.565 (+)** | 76.27 | 2.786 | **5.494** | 72.70 |
| ***Dungeness Crab*** | 1.953 | | **5.225** | | 80.325 | | 1.628 | | **3.235** | 79.50 | 2.063 | **4.068** | 76.768 |
| **Pacific Hake** | 0.9392 | | **2.513** | | 82.838 | | 1.15 | | **2.284** | 81.784 | 0.9555 | **1.884** | 78.652 |
| ***Ocean Shrimp*** | 0.8253 | | **2.208 (+)** | | 85.046 | | 0.5479 | | **1.089** | 82.873 | 0.4998 | 0.9855 | 79.6375 |
| ***Chub Mackerel*** | 0.6888 | | **1.843 (+)** | | 86.889 | | 0.9958 | | **1.978** | 84.851 | 1.283 | **2.529** | 82.1665 |
| ***Pacific Herring*** | 0.4844 | | **1.296 (+)** | | 88.185 | | 0.352 | | 0.6994 | 85.5504 | 0.4499 | 0.8872 | 83.0537 |
| **Sea Urchin *(Strongylocentrotus)*** | 0.3602 | | 0.9639 | | 89.1489 | | 1.576 | | **3.13** | 88.6804 | <0.001 | <0.001 | 83.0537 |
| ***Chinook Salmon*** | 0.315 | | 0.8428 | | 89.9917 | | 0.4031 | | 0.8008 | 89.4812 | 0.68 | **1.341** | 84.3947 |
| ***Yellowfin Tuna (tunas)*** | 0.2295 | | 0.6141 | | 90.6058 | | 0.4327 | | 0.8597 | 90.3409 | 0.2753 | 0.5428 | 84.9375 |
| ***Pacific Saury*** | <0.001 | | <0.001 | | 90.6058 | | <0.001 | | <0.001 | 90.3409 | 2.394 | **4.722 (+)** | 89.6595 |
| ***Pacific Oyster*** | 0.0735 | | 0.1968 | | 90.8026 | | 0.1559 | | 0.3096 | 90.6505 | 0.8298 | **1.636** | 91.2955 |
| ***California Spiny Lobster (lobsters)*** | 0.0169 | | 0.0453 | | 90.8479 | | 0.0168 | | 0.0335 | 90.6840 | <0.001 | <0.001 | 91.2955 |
| ***Bigeye Tuna*** | 0.1106 | | 0.2960 | | 91.1439 | | 0.2256 | | 0.4482 | 91.1322 | 0.2052 | 0.4046 | 91.7001 |
| ***Spot Shrimp (commercial prawns)*** | 0.0269 | | 0.0720 | | 91.2159 | | 0.0309 | | 0.0615 | 91.1937 | 0.0057 | 0.0113 | 91.7114 |
| **Sablefish** | 0.1265 | | 0.3386 | | 91.5545 | | 0.1644 | | 0.3267 | 91.5204 | 0.1471 | 0.2901 | 92.0015 |
| California Halibut | 0.0613 | | 0.1640 | | 91.7185 | | 0.0553 | | 0.1100 | 91.6304 | <0.001 | <0.001 | 92.0015 |
| Swordfish | 0.0639 | | 0.1712 | | 91.8897 | | 0.0815 | | 0.1620 | 91.7924 | 0.1555 | 0.3066 | 92.3081 |
| Albacore Tuna | 0.169 | | 0.4522 | | 92.3419 | | 0.1751 | | 0.3480 | 92.1404 | 0.2339 | 0.4613 | 92.7694 |
| Hagfishes | 0.1104 | | 0.2955 | | 92.6374 | | 0.1748 | | 0.3473 | 92.4877 | <0.001 | <0.001 | 92.7694 |
| Shortspine Thornyhead | 0.0210 | | 0.0563 | | 92.6937 | | 0.0663 | | 0.1319 | 92.6196 | 0.0620 | 0.1223 | 92.8917 |
| Sea Cucumber | 0.0467 | | 0.1252 | | 92.8189 | | 0.0872 | | 0.1733 | 92.7929 | <0.001 | <0.001 | 92.8917 |
| Red Rock Crab | 0.1468 | | 0.3929 | | 93.2118 | | 0.0432 | | 0.0859 | 92.8788 | 0.0490 | 0.0968 | 92.9885 |
| Pacific Bluefin Tuna | 0.0691 | | 0.1849 | | 93.3967 | | 0.0974 | | 0.1935 | 93.0723 | 0.1091 | 0.2152 | 93.2037 |
| Pacific Rock Shrimp | 0.0786 | | 0.2103 | | 93.6070 | | 0.0466 | | 0.0926 | 93.1649 | 0.0272 | 0.0538 | 93.2575 |
| **California (cont’d)^[[4]](#footnote-4)^** | **Revenue** | | | | | |  |  |  |  |  |  |  |
|  | **Pre-Heatwave v. Post-Heatwave** | | | | | |  |  |  |  |  |  |  |
| **Taxon** | **Av. dissim** | | **Contrib. %** | | **Cumul. %** | |  |  |  |  |  |  |  |
| **California Market Squid** | 6.671 | | **20.79** | | 20.79 | |  |  |  |  |  |  |  |
| **Pacific Sardine** | 1.41 | | **4.396** | | 25.186 | |  |  |  |  |  |  |  |
| ***Northern Anchovy*** | 0.1507 | | <0.001 | | 25.186 | |  |  |  |  |  |  |  |
| ***Dungeness Crab*** | 7.384 | | **23.01 (+)** | | 48.196 | |  |  |  |  |  |  |  |
| **Pacific Hake** | 0.1222 | | <0.001 | | 48.196 | |  |  |  |  |  |  |  |
| ***Ocean Shrimp*** | 0.4698 | | **1.464** | | 49.66 | |  |  |  |  |  |  |  |
| ***Chub Mackerel*** | 0.1173 | | <0.001 | | 49.66 | |  |  |  |  |  |  |  |
| ***Pacific Herring*** | 0.1201 | | <0.001 | | 49.66 | |  |  |  |  |  |  |  |
| **Sea Urchin (*Strongylocentrotus*)** | 0.5254 | | **1.638** | | 51.298 | |  |  |  |  |  |  |  |
| ***Chinook Salmon*** | 2.151 | | **6.703 (+)** | | 58.001 | |  |  |  |  |  |  |  |
| ***Yellowfin Tuna (tunas)*** | 0.4536 | | **1.414 (+)** | | 59.415 | |  |  |  |  |  |  |  |
| ***Pacific Saury*** | <0.001 | | <0.001 | | 59.415 | |  |  |  |  |  |  |  |
| ***Pacific Oyster*** | 3.566 | | **11.11 (+)** | | 70.525 | |  |  |  |  |  |  |  |
| ***California Spiny Lobster (lobsters)*** | 1.287 | | **4.011 (+)** | | 74.536 | |  |  |  |  |  |  |  |
| ***Bigeye Tuna*** | 1.067 | | **3.325 (+)** | | 77.861 | |  |  |  |  |  |  |  |
| ***Spot Shrimp (commercial prawns)*** | 0.9645 | | **3.006 (+)** | | 80.867 | |  |  |  |  |  |  |  |
| **Sablefish** | 0.9008 | | **2.807** | | 83.674 | |  |  |  |  |  |  |  |
| California Halibut | 0.2931 | | 0.9134 | | 84.5874 | |  |  |  |  |  |  |  |
| Swordfish | 0.2799 | | 0.8723 | | 85.4597 | |  |  |  |  |  |  |  |
| Albacore Tuna | 0.2427 | | 0.7565 | | 86.2162 | |  |  |  |  |  |  |  |
| Hagfishes | 0.2293 | | 0.7146 | | 86.9308 | |  |  |  |  |  |  |  |
| Shortspine Thornyhead | 0.2223 | | 0.6929 | | 87.6237 | |  |  |  |  |  |  |  |
| Sea Cucumber | 0.2056 | | 0.6409 | | 88.2646 | |  |  |  |  |  |  |  |
| Red Rock Crab | 0.1978 | | 0.6164 | | 88.881 | |  |  |  |  |  |  |  |
| Pacific Bluefin Tuna | 0.1926 | | 0.6003 | | 89.4813 | |  |  |  |  |  |  |  |
| Pacific Rock Shrimp | 0.1723 | | 0.5371 | | 90.0184 | |  |  |  |  |  |  |  |
| **Pacific NW^[[5]](#footnote-5)^** | **Biomass** | | | | | **Landings** | | | | | | | |
|  | **Pre-Heatwave v. Post-Heatwave** | | | | | **Pre-Heatwave v. Post-Heatwave** | | | | | **Heatwave v. Post-Heatwave** | | |
| **Taxon** | **Av. dissim** | **Contrib. %** | | **Cumul. %** | | **Av. dissim** | | **Contrib. %** | | **Cumul. %** | **Av. dissim** | **Contrib. %** | **Cumul. %** |
| ***North Pacific Hake*** | 11.52 | **40.48 (+)** | | 40.48 | | 12.24 | | **35.75 (+)** | | 35.75 | 10.18 | **36.93 (+)** | 36.93 |
| **Pacific Sardine** | 4.335 | **15.23** | | 55.71 | | 10.04 | | **29.32** | | 65.07 | 2.101 | **7.619** | 44.549 |
| ***Pacific Oyster*** | 2.36 | **8.294** | | 64.004 | | 0.2834 | | 0.8277 | | 65.8977 | 0.2056 | 0.7458 | 45.2948 |
| ***Sockeye Salmon*** | 1.948 | **6.845 (+)** | | 70.849 | | 0.3071 | | 0.8967 | | 66.7944 | 0.2791 | **1.012** | 46.3068 |
| ***Ocean Shrimp*** | 0.66 | **2.319 (+)** | | 73.168 | | 1.935 | | **5.649 (+)** | | 72.4434 | 5.179 | **18.78** | 65.0868 |
| **Albacore Tuna** | 0.6481 | **2.277** | | 75.445 | | 0.911 | | **2.66** | | 75.1034 | 1.086 | **3.937** | 69.0238 |
| ***Dungeness Crab*** | 0.5898 | **2.073** | | 77.518 | | 0.8566 | | **2.501** | | 77.6044 | 2.097 | **7.607 (+)** | 76.6308 |
| ***Widow Rockfish*** | 0.5776 | **2.03 (+)** | | 79.548 | | 1.634 | | **4.772 (+)** | | 82.3764 | 1.514 | **5.491 (+)** | 82.1218 |
| ***Pacific Jack Mackerel*** | 0.4761 | **1.673 (+)** | | 81.221 | | 0.0551 | | 0.1609 | | 82.5373 | 0.1064 | 0.3861 | 82.5079 |
| **Pink Salmon** | 0.4462 | **1.568** | | 82.789 | | 0.6869 | | 2.006 | | 84.5433 | 0.1803 | 0.6541 | 83.162 |
| **Chum Salmon** | 0.4074 | **1.432** | | 84.221 | | 0.6564 | | **1.917** | | 86.4603 | 0.5543 | **2.01** | 85.172 |
| ***Flatfishes*** | 0.3094 | **1.087 (+)** | | 85.308 | | 0.0033 | | 0.0097 | | 86.4700 | 0.0037 | 0.0135 | 85.1855 |
| ***Venus Clams (clams)*** | 0.2324 | 0.8167 | | 86.1247 | | 0.0480 | | 0.1403 | | 86.6103 | 0.0806 | 0.2926 | 85.4781 |
| **Chinook Salmon** | 0.2043 | 0.718 | | 86.8427 | | 0.3021 | | 0.8821 | | 87.4924 | 0.7963 | **2.888** | 88.3661 |
| Yellowfin Tuna | 0.1799 | 0.6321 | | 87.4748 | | <0.001 | | <0.001 | | 87.4924 | <0.001 | <0.001 | 88.3661 |
| Pacific Herring | 0.1735 | 0.6098 | | 88.0846 | | 0.0232 | | 0.0680 | | 87.5604 | 0.0225 | 0.0817 | 88.4478 |
| ***California Market Squid*** | 0.0529 | 0.1861 | | 88.2707 | | 0.5794 | | **1.692 (+)** | | 89.2524 | 0.594 | **2.155 (+)** | 90.6028 |
| ***Yellowtail Rockfish*** | 0.1297 | 0.4558 | | 88.7265 | | 0.5213 | | **1.522 (+)** | | 90.7744 | 0.3237 | **1.174 (+)** | 91.7768 |
| **Dover Sole** | 0.1603 | 0.5634 | | 89.2899 | | 0.4647 | | **1.357** | | 92.1314 | 0.208 | 0.7544 | 92.5312 |
| **Arrowtooth Flounder** | 0.1636 | 0.5748 | | 89.8647 | | 0.4559 | | **1.331** | | 93.4624 | 0.1399 | 0.5076 | 93.0388 |
| **Coho Salmon** | 0.0863 | 0.3034 | | 90.1681 | | 0.2704 | | 0.7897 | | 94.2521 | 0.3037 | **1.101** | 94.1398 |
| **Sablefish** | 0.1672 | 0.5877 | | 90.7558 | | 0.1908 | | 0.5572 | | 94.8093 | 0.1802 | 0.6536 | 94.7934 |
| ***Petrale Sole*** | 0.0715 | 0.2516 | | 91.0074 | | 0.2068 | | 0.6037 | | 95.4130 | 0.0804 | 0.2919 | 95.0853 |
| ***Pacific Geoduck Clam*** | 0.1331 | 0.4676 | | 91.475 | | 0.0309 | | 0.0903 | | 95.5033 | 0.0277 | 0.1006 | 95.1859 |

| **Pacific NW (cont’d)^[[6]](#footnote-6)^** | **Revenue** | | |
| --- | --- | --- | --- |
|  | **Pre-Heatwave v. Post-Heatwave** | | |
| **Taxon** | **Av. dissim** | **Contrib. %** | **Cumul. %** |
| ***North Pacific Hake*** | 1.394 | **5.742 (+)** | 5.742 |
| **Pacific Sardine** | 0.9944 | **4.097** | 9.839 |
| ***Pacific Oyster*** | 1.175 | **4.839 (+)** | 14.678 |
| ***Sockeye Salmon*** | 0.5302 | **2.184** | 16.862 |
| ***Ocean Shrimp*** | 1.86 | **7.661 (+)** | 24.523 |
| **Albacore Tuna** | 1.064 | **4.383** | 28.906 |
| ***Dungeness Crab*** | 8.014 | **33.02 (+)** | 61.926 |
| ***Widow Rockfish*** | 0.4845 | **1.996 (+)** | 63.922 |
| ***Pacific Jack Mackerel*** | 0.0014 | 0.0057 | 63.9277 |
| **Pink Salmon** | 0.2803 | **1.155** | 65.0827 |
| **Chum Salmon** | 0.5116 | **2.108** | 67.1907 |
| ***Flatfishes*** | 0.0013 | 0.0053 | 67.1960 |
| ***Venus Clams (clams)*** | 0.9073 | **3.738 (+)** | 70.9340 |
| **Chinook Salmon** | 0.7275 | **2.997** | 73.9310 |
| Yellowfin Tuna | <0.001 | <0.001 | 73.9310 |
| Pacific Herring | 0.0115 | 0.0473 | 73.9783 |
| ***California Market Squid*** | 0.359 | **1.479 (+)** | 75.4573 |
| ***Yellowtail Rockfish*** | 0.1339 | 0.5518 | 76.0091 |
| **Dover Sole** | 0.1714 | 0.7063 | 76.7154 |
| **Arrowtooth Flounder** | 0.0569 | 0.2345 | 76.9499 |
| **Coho Salmon** | 0.2582 | **1.064** | 78.0139 |
| **Sablefish** | 0.9151 | **3.77** | 81.7839 |
| ***Petrale Sole*** | 0.3111 | **1.282 (+)** | 83.0659 |
| ***Pacific Geoduck Clam*** | 2.399 | **9.882 (+)** | 92.9479 |

| **Gulf of Maine^[[7]](#footnote-7)^** | **Biomass** | | |
| --- | --- | --- | --- |
|  | **Pre-Accel v. Post-Accel/Post-Heatwave** | | |
| **Taxon** | **Av. dissim** | **Contrib. %** | **Cumul. %** |
| **American Sea Scallop** | 7.2330 | **21.760** | 21.76 |
| ***Atlantic Surf Clam*** | 2.5000 | **7.521 (+)** | 29.28 |
| ***Ocean Quahog*** | 2.3020 | **6.926 (+)** | 36.21 |
| ***American Lobster*** | 2.2380 | **6.735 (+)** | 42.94 |
| **Clams** | 2.2230 | **6.688** | 49.63 |
| ***Atlantic Herring*** | 1.8350 | **5.521** | 55.15 |
| ***Shortfin Squid*** | 1.5510 | **4.665 (+)** | 59.82 |
| ***Atlantic Mackerel*** | 1.0340 | **3.111** | 62.93 |
| ***Atlantic Cod*** | 0.9243 | **2.781** | 65.71 |
| ***Haddock*** | 0.7936 | **2.388 (+)** | 68.10 |
| **Northern Quahog** | 0.7253 | **2.182** | 70.28 |
| **Goosefish** | 0.6479 | **1.949** | 72.23 |
| **Yellowtail Flounder** | 0.6262 | **1.884** | 74.11 |
| **Silver Hake** | 0.4985 | **1.500** | 75.61 |
| **Sea Urchin (*Strongylocentrotus*)** | 0.4395 | **1.322** | 76.94 |
| ***Skates and Rays*** | 0.4358 | **1.311** | 78.25 |
| **Crustaceans** | 0.4339 | **1.306** | 79.55 |
| **Mollusks** | 0.4194 | **1.262** | 80.81 |
| ***Jonah Crab*** | 0.3920 | **1.180 (+)** | 81.99 |
| **Longfin inshore squid** | 0.3431 | **1.032** | 83.03 |
| Bluefish | 0.3291 | 0.990 | 84.02 |
| Little Skate | 0.2431 | 0.732 | 84.75 |
| Atlantic Butterfish | 0.2383 | 0.717 | 85.46 |
| Scup | 0.2313 | 0.696 | 86.16 |
| Squids | 0.2292 | 0.690 | 86.85 |
| ***Saithe (Pollock)*** | 0.2229 | 0.671 | 87.52 |
| ***Piked (Spiny) Dogfish*** | 0.2145 | 0.645 | 88.17 |
| **Witch Flounder** | 0.2127 | 0.640 | 88.81 |
| Blue Shark | 0.2040 | 0.614 | 89.42 |
| ***Acadian Redfish*** | 0.0874 | 0.263 | 89.68 |
| ***Winter Skate*** | 0.0000 | 0.000 | 89.68 |
| ***Menhadens*** | 0.0017 | 0.005 | 89.69 |
| ***Rockweed Seaweed*** | 0.0000 | 0.000 | 89.69 |
| **Crabs** | 0.1823 | 0.549 | 90.24 |
| **Winter Flounder** | 0.1549 | 0.466 | 90.70 |
| **Sea Cucumbers** | 0.0000 | 0.000 | 90.70 |
| ***Striped Bass*** | 0.1458 | 0.439 | 91.14 |
| **Rockweeds** | 0.0000 | 0.000 | 91.14 |
| American Plaice Flounder | 0.2011 | 0.605 | 91.75 |
| ***Northern Shrimp*** | 0.0835 | 0.251 | 92.00 |
| ***Bluefish*** | 0.3291 | 0.990 | 92.99 |
| Hagfishes | 0.0731 | 0.220 | 93.21 |
| ***Eastern Oyster*** | 0.1891 | 0.569 | 93.78 |
| ***Soft Clam*** | 0.0000 | 0.000 | 93.78 |
| ***American Eel*** | 0.0017 | 0.005 | 93.78 |
| Bluefin Tuna | 0.0531 | 0.160 | 93.94 |
| Green Sea Urchin | 0.0453 | 0.136 | 94.08 |

| **Gulf of Maine (cont’d)^[[8]](#footnote-8)^** | **Landings** | | | | | | | | |
| --- | --- | --- | --- | --- | --- | --- | --- | --- | --- |
|  | **Pre-Accel v. Post-Accel/Post-Heatwave** | | | **Pre-Accel v. Accel/Pre-Heatwave** | | | **Post-Accel/Post-Heatwave v. Accel/Pre-Heatwave** | | |
| **Taxon** | **Av. dissim** | **Contrib. %** | **Cumul. %** | **Av. dissim** | **Contrib. %** | **Cumul. %** | **Av. dissim** | **Contrib. %** | **Cumul. %** |
| ***American Sea Scallop*** | 0.9126 | **2.317 (+)** | 2.32 | 0.6617 | **2.590 (+)** | 2.59 | 0.6296 | **2.356 (+)** | 2.36 |
| ***Atlantic Surf Clam*** | 1.4950 | **3.795 (+)** | 6.11 | 0.5450 | **2.134 (+)** | 4.72 | 0.9130 | **3.417** | 5.77 |
| ***Ocean Quahog*** | 0.8380 | **2.128** | 8.24 | 0.7975 | **3.122 (+)** | 7.85 | 0.8597 | **3.218 (+)** | 8.99 |
| ***American Lobster*** | 6.0480 | **15.360 (+)** | 23.60 | 3.0700 | **12.020 (+)** | 19.87 | 2.7640 | **10.350** | 19.34 |
| **Clams** | 0.4925 | **1.251** | 24.85 | 0.4609 | **1.804** | 21.67 | 0.0000 | 0.000 | 19.34 |
| ***Atlantic Herring*** | 6.8560 | **17.410** | 42.26 | 3.1480 | **12.320 (+)** | 33.99 | 6.8720 | **25.720 (+)** | 45.06 |
| ***Shortfin Squid*** | 0.5375 | **1.365 (+)** | 43.63 | 0.0107 | 0.042 | 34.03 | 0.5296 | **1.982** | 47.04 |
| ***Atlantic Mackerel*** | 2.6530 | **6.736** | 50.36 | 2.4400 | **9.550** | 43.58 | 0.9980 | **3.735 (+)** | 50.78 |
| ***Atlantic Cod*** | 2.1180 | **5.378** | 55.74 | 0.5035 | **1.971** | 45.55 | 1.9340 | **7.238 (+)** | 58.02 |
| ***Haddock*** | 0.5770 | **1.465 (+)** | 57.21 | 0.5161 | **2.021 (+)** | 47.57 | 0.5478 | **2.050 (+)** | 60.07 |
| **Northern Quahog** | 0.0702 | 0.178 | 57.38 | 0.0822 | 0.322 | 47.90 | 0.0295 | 0.110 | 60.18 |
| **Goosefish** | 1.5360 | **3.901** | 61.28 | 1.7330 | **6.785** | 54.68 | 0.3458 | **1.294** | 61.47 |
| **Yellowtail Flounder** | 0.7499 | **1.904** | 63.19 | 0.5360 | **2.098** | 56.78 | 0.1772 | **0.663** | 62.13 |
| **Silver Hake** | 0.2140 | 0.544 | 63.73 | 0.2155 | 0.844 | 57.62 | 0.2036 | 0.762 | 62.90 |
| **Sea Urchin (*Strongylocentrotus*)** | 0.6337 | **1.609** | 65.34 | 0.5918 | **2.317** | 59.94 | 0.0025 | 0.009 | 62.90 |
| ***Skates and Rays*** | 1.6450 | **4.176** | 69.52 | 0.7072 | **2.769** | 62.71 | 1.0900 | **4.080 (+)** | 66.98 |
| **Crustaceans** | 0.2373 | 0.603 | 70.12 | 0.2221 | 0.870 | 63.58 | 0.0000 | 0.000 | 66.98 |
| **Mollusks** | 0.0000 | 0.000 | 70.12 | 0.0000 | 0.000 | 63.58 | 0.0000 | 0.000 | 66.98 |
| ***Jonah Crab*** | 0.7083 | **1.799 (+)** | 71.92 | 0.2167 | 0.848 | 64.43 | 0.4734 | **1.772** | 68.76 |
| **Longfin inshore squid** | 0.0946 | 0.240 | 72.16 | 0.0765 | 0.299 | 64.73 | 0.0825 | 0.309 | 69.07 |
| Bluefish | 0.2528 | 0.642 | 72.80 | 0.2528 | 0.990 | 65.72 | 0.4187 | 1.567 | 70.63 |
| Little Skate | 0.0481 | 0.122 | 72.92 | 0.1629 | 0.638 | 66.35 | 0.1340 | 0.501 | 71.13 |
| Atlantic Butterfish | 0.0245 | 0.062 | 72.98 | 0.0127 | 0.050 | 66.40 | 0.0131 | 0.049 | 71.18 |
| Scup | 0.1753 | 0.445 | 73.43 | 0.1521 | 0.596 | 67.00 | 0.1212 | 0.454 | 71.64 |
| Squids | 0.0023 | 0.006 | 73.44 | 0.0021 | 0.008 | 67.01 | 0.0000 | 0.000 | 71.64 |
| ***Saithe (Pollock)*** | 0.5911 | **1.501 (+)** | 74.94 | 0.5382 | **2.107 (+)** | 69.11 | 0.9689 | **3.626 (+)** | 75.26 |
| ***Piked (Spiny) Dogfish*** | 0.5065 | **1.286 (+)** | 76.22 | 0.4575 | **1.791 (+)** | 70.90 | 0.2303 | 0.862 | 76.12 |
| **Witch Flounder** | 0.3385 | 0.859 | 77.08 | 0.2695 | **1.055** | 71.96 | 0.0528 | 0.197 | 76.32 |
| Blue Shark | 0.0049 | 0.012 | 77.09 | 0.0046 | 0.018 | 71.98 | 0.0000 | 0.000 | 76.32 |
| ***Acadian Redfish*** | 0.9270 | **2.354 (+)** | 79.45 | 0.2408 | 0.943 | 72.92 | 0.6624 | **2.479** | 78.80 |
| ***Winter Skate*** | 0.9015 | **2.289 (+)** | 81.74 | 0.4738 | **1.855 (+)** | 74.78 | 0.4815 | **1.802** | 80.60 |
| ***Menhadens*** | 0.8441 | **2.143 (+)** | 83.88 | 0.4223 | **1.653 (+)** | 76.43 | 0.6938 | **2.597** | 83.20 |
| ***Rockweed Seaweed*** | 0.7268 | **1.845 (+)** | 85.73 | 0.9196 | **3.600 (+)** | 80.03 | 0.9534 | **3.568 (+)** | 86.77 |
| **Crabs** | 0.6622 | **1.681** | 87.41 | 0.6188 | **2.423** | 82.45 | 0.0501 | 0.187 | 86.96 |
| **Winter Flounder** | 0.5274 | **1.339** | 88.75 | 0.3437 | **1.345** | 83.80 | 0.2026 | 0.758 | 87.71 |
| **Sea Cucumbers** | 0.4722 | **1.199** | 89.94 | 0.4445 | **1.740** | 85.54 | 0.0175 | 0.066 | 87.78 |
| ***Striped Bass*** | 0.3704 | 0.941 | 90.88 | 0.2911 | **1.140 (+)** | 86.68 | 0.5667 | **2.121 (+)** | 89.90 |
| **Rockweeds** | 0.3415 | 0.867 | 91.75 | 0.0000 | 0.000 | 86.68 | 0.3382 | **1.266** | 91.17 |
| American Plaice Flounder | 0.2877 | 0.731 | 92.48 | 0.2374 | 0.929 | 87.61 | 0.0666 | 0.249 | 91.42 |
| ***Northern Shrimp*** | 0.1765 | 0.448 | 92.93 | 0.8036 | **3.146 (+)** | 90.75 | 0.9672 | **3.620 (+)** | 95.04 |
| ***Bluefish*** | 0.2528 | 0.642 | 93.57 | 0.2528 | 0.990 | 91.74 | 0.4187 | **1.567 (+)** | 96.60 |
| Hagfishes | 0.2202 | 0.559 | 94.13 | 0.2066 | 0.809 | 92.55 | 0.0405 | 0.152 | 96.75 |
| ***Eastern Oyster*** | 0.0577 | 0.146 | 94.28 | 0.0161 | 0.063 | 92.61 | 0.0400 | 0.150 | 96.90 |
| ***Soft Clam*** | 0.0637 | 0.162 | 94.44 | 0.0617 | 0.241 | 92.85 | 0.0763 | 0.286 | 97.19 |
| ***American Eel*** | 0.0016 | 0.004 | 94.44 | 0.0014 | 0.005 | 92.86 | 0.0007 | 0.003 | 97.19 |
| Bluefin Tuna | 0.1496 | 0.380 | 94.82 | 0.1035 | 0.405 | 93.26 | 0.1133 | 0.424 | 97.62 |
| Green Sea Urchin | 0.1529 | 0.388 | 95.21 | 0.1471 | 0.576 | 93.84 | 0.1078 | 0.404 | 98.02 |

| **Gulf of Maine (cont’d)^[[9]](#footnote-9)^** | **Revenue** | | | | | |
| --- | --- | --- | --- | --- | --- | --- |
|  | **Pre-Accel v. Post-Accel/Post-Heatwave** | | | **Post-Accel/Post-Heatwave v. Accel/Pre-Heatwave** | | |
| **Taxon** | **Av. dissim** | **Contrib. %** | **Cumul. %** | **Av. dissim** | **Contrib. %** | **Cumul. %** |
| ***American Sea Scallop*** | 10.0700 | **26.730 (+)** | 26.73 | 4.1360 | **18.910 (+)** | 18.91 |
| ***Atlantic Surf Clam*** | 0.8343 | **2.215 (+)** | 28.95 | 0.4899 | **2.240 (+)** | 21.15 |
| ***Ocean Quahog*** | 0.2683 | 0.712 | 29.66 | 0.2584 | **1.181** | 22.33 |
| ***American Lobster*** | 14.7400 | **39.140 (+)** | 68.80 | 10.6100 | **48.500 (+)** | 70.83 |
| **Clams** | 0.1469 | 0.390 | 69.19 | 0.0000 | 0.000 | 70.83 |
| ***Atlantic Herring*** | 1.1730 | **3.114** | 72.30 | 0.3029 | **1.385** | 72.22 |
| ***Shortfin Squid*** | 0.1044 | 0.277 | 72.58 | 0.0952 | 0.435 | 72.65 |
| ***Atlantic Mackerel*** | 0.1435 | 0.381 | 72.96 | 0.0872 | 0.399 | 73.05 |
| ***Atlantic Cod*** | 1.0790 | **2.865** | 75.82 | 1.0920 | **4.992** | 78.04 |
| ***Haddock*** | 0.2618 | 0.695 | 76.52 | 0.2706 | **1.237** | 79.28 |
| **Northern Quahog** | 0.2552 | 0.678 | 77.20 | 0.0721 | 0.330 | 79.61 |
| **Goosefish** | 0.8190 | **2.174** | 79.37 | 0.1141 | 0.522 | 80.13 |
| **Yellowtail Flounder** | 0.4539 | **1.205** | 80.58 | 0.1212 | 0.554 | 80.68 |
| **Silver Hake** | 0.1558 | 0.414 | 80.99 | 0.0733 | 0.335 | 81.02 |
| **Sea Urchin (*Strongylocentrotus*)** | 0.4827 | **1.282** | 82.27 | 0.0030 | 0.014 | 81.03 |
| ***Skates and Rays*** | 0.1569 | 0.417 | 82.69 | 0.1181 | 0.540 | 81.57 |
| **Crustaceans** | 0.1485 | 0.394 | 83.08 | 0.0000 | 0.000 | 81.57 |
| **Mollusks** | 0.0000 | 0.000 | 83.08 | 0.0000 | 0.000 | 81.57 |
| ***Jonah Crab*** | 0.4067 | **1.080 (+)** | 84.16 | 0.2964 | **1.355 (+)** | 82.93 |
| **Longfin inshore squid** | 0.0757 | 0.201 | 84.36 | 0.0689 | 0.315 | 83.24 |
| Bluefish | 0.0119 | 0.032 | 84.40 | 0.0077 | 0.035 | 83.28 |
| Little Skate | 0.0035 | 0.009 | 84.41 | 0.0077 | 0.035 | 83.31 |
| Atlantic Butterfish | 0.0100 | 0.027 | 84.43 | 0.0055 | 0.025 | 83.34 |
| Scup | 0.0403 | 0.107 | 84.54 | 0.0211 | 0.097 | 83.43 |
| Squids | 0.0007 | 0.002 | 84.54 | 0.0000 | 0.000 | 83.43 |
| ***Saithe (Pollock)*** | 0.1178 | 0.313 | 84.85 | 0.1752 | 0.801 | 84.24 |
| ***Piked (Spiny) Dogfish*** | 0.0586 | 0.156 | 85.01 | 0.0302 | 0.138 | 84.37 |
| **Witch Flounder** | 0.2313 | 0.614 | 85.62 | 0.0468 | 0.214 | 84.59 |
| Blue Shark | 0.0000 | 0.000 | 85.62 | 0.0000 | 0.000 | 84.59 |
| ***Acadian Redfish*** | 0.2795 | 0.742 | 86.36 | 0.1838 | 0.840 | 85.43 |
| ***Winter Skate*** | 0.1338 | 0.355 | 86.72 | 0.0736 | 0.337 | 85.76 |
| ***Menhadens*** | 0.1047 | 0.278 | 87.00 | 0.0885 | 0.405 | 86.17 |
| ***Rockweed Seaweed*** | 0.0171 | 0.045 | 87.04 | 0.0213 | 0.097 | 86.27 |
| **Crabs** | 0.2173 | 0.577 | 87.62 | 0.0100 | 0.046 | 86.31 |
| **Winter Flounder** | 0.1964 | 0.521 | 88.14 | 0.1013 | 0.463 | 86.78 |
| **Sea Cucumbers** | 0.0187 | 0.050 | 88.19 | 0.0031 | 0.014 | 86.79 |
| ***Striped Bass*** | 0.0785 | 0.208 | 88.40 | 0.0361 | 0.165 | 86.95 |
| **Rockweeds** | 0.0236 | 0.063 | 88.46 | 0.0215 | 0.098 | 87.05 |
| American Plaice Flounder | 0.1398 | 0.371 | 88.83 | 0.0496 | 0.227 | 87.28 |
| ***Northern Shrimp*** | 0.0488 | 0.130 | 88.96 | 0.2847 | **1.301** | 88.58 |
| ***Bluefish*** | 0.0119 | 0.032 | 88.99 | 0.0077 | 0.035 | 88.62 |
| Hagfishes | 0.0422 | 0.112 | 89.11 | 0.0213 | 0.097 | 88.71 |
| ***Eastern Oyster*** | 1.3970 | **3.710 (+)** | 92.82 | 0.9259 | **4.233 (+)** | 92.95 |
| ***Soft Clam*** | 0.4109 | **1.091 (+)** | 93.91 | 0.1915 | 0.876 | 93.82 |
| ***American Eel*** | 0.3837 | **1.019 (+)** | 94.93 | 0.3757 | **1.718 (+)** | 95.54 |
| Bluefin Tuna | 0.3203 | 0.850 | 95.78 | 0.1305 | 0.597 | 96.14 |
| Green Sea Urchin | 0.2543 | 0.675 | 96.45 | 0.1332 | 0.609 | 96.75 |

| **Gulf of Mexico^[[10]](#footnote-10)^** | **Biomass** | | | | | | **Landings** | | |
| --- | --- | --- | --- | --- | --- | --- | --- | --- | --- |
|  | **Pre-Katrina v. Post-Katrina/Pre-DWH** | | | **Pre-Katrina v. Post-DWH** | | | **Pre-Katrina v. Post-DWH** | | |
| **Taxon** | **Av. dissim** | **Contrib. %** | **Cumul. %** | **Av. dissim** | **Contrib. %** | **Cumul. %** | **Av. dissim** | **Contrib. %** | **Cumul. %** |
| **Gulf Menhaden** | 4.445 | **41.2** | 41.2 | 4.259 | **31.32** | 31.32 | 0.0156 | 0.1324 | 0.1324 |
| ***Eastern Oyster*** | 0.9134 | **8.466** | 49.666 | 2.329 | **17.12** | 48.44 | **0.2037** | **1.721** | 1.8534 |
| ***White Shrimp*** | 0.7112 | **6.591 (+)** | 56.257 | 0.4551 | **3.347 (+)** | 51.787 | **0.531** | **4.488 (+)** | 6.3414 |
| **Atlantic Croaker** | 0.5414 | **5.018** | 61.275 | 0.7865 | **5.784** | 57.571 | 0.0130 | 0.1106 | 6.4520 |
| ***Misc. Crustaceans*** | 0.425 | **3.939 (+)** | 65.214 | 0.4091 | **3.008 (+)** | 60.579 | 0.0069 | 0.0591 | 6.5111 |
| **Spotted Seatrout** | 0.2128 | **1.972** | 67.186 | 0.2128 | **1.972** | 62.551 | **0.4281** | **3.618** | 10.1291 |
| **Weakfishes** | 0.1981 | **1.836** | 69.022 | 0.2678 | **1.97** | 64.521 | <0.001 | <0.001 | 10.1291 |
| ***Blue Crab*** | 0.1797 | **1.665** | 70.687 | 0.274 | **2.015** | 66.536 | **0.2218** | **1.874** | 12.0031 |
| **Striped Mullet** | 0.1747 | **1.619** | 72.306 | 0.1124 | 0.8269 | 67.3629 | **0.2098** | **1.773** | 13.7761 |
| **Porgies** | 0.1707 | **1.582** | 73.888 | 0.2769 | **2.037** | 69.3999 | <0.001 | 0.0016 | 13.7777 |
| **Gag** | 0.1469 | **1.361** | 75.249 | 0.2675 | **1.967** | 71.3669 | 0.0043 | 0.0366 | 13.8143 |
| **Atlantic Sea Bob** | 0.1287 | **1.193** | 76.442 | 0.1297 | 0.9535 | 72.3204 | **0.2036** | **1.721** | 15.5353 |
| ***Red Snapper*** | 0.113 | **1.047** | 77.489 | 0.0777 | 0.5721 | 72.8925 | 0.0619 | 0.5236 | 16.0589 |
| Dolphinfish | 0.0973 | 0.9019 | 78.3909 | 0.1119 | 0.8232 | 73.7157 | 0.0088 | 0.0745 | 16.1334 |
| **Sheepshead** | 0.0894 | 0.829 | 79.2199 | 0.1624 | **1.195** | 74.9107 | **0.1536** | **1.298** | 17.4314 |
| Calico Scallop | 0.0747 | 0.693 | 79.9129 | 0.0745 | 0.5482 | 75.4589 | <0.001 | <0.001 | 17.4314 |
| **Red Drum** | 0.0726 | 0.6732 | 80.5861 | 0.3626 | **2.667** | 78.1259 | **0.4976** | **4.205** | 21.6364 |
| Red Grouper | 0.0707 | 0.6556 | 81.2417 | 0.0690 | 0.5075 | 78.6334 | <0.001 | <0.001 | 21.6364 |
| Spanish Mackerel | 0.0638 | 0.5914 | 81.8331 | 0.0719 | 0.5291 | 79.1625 | 0.0063 | 0.0535 | 21.6899 |
| Mullets | 0.0608 | 0.5641 | 82.3972 | 0.0488 | 0.3592 | 79.5217 | <0.001 | 0.0049 | 21.6948 |
| Northern Quahog | 0.0586 | 0.5431 | 82.9403 | 0.0769 | 0.5661 | 80.0878 | <0.001 | <0.001 | 21.6948 |
| Rock Shrimp | 0.0574 | 0.5322 | 83.4725 | 0.0492 | 0.3622 | 80.4500 | 0.0201 | 0.1699 | 21.8647 |
| Vermilion Snapper | 0.0496 | 0.4602 | 83.9327 | 0.0296 | 0.2183 | 80.6683 | 0.0165 | 0.14 | 22.0047 |
| ***Brown Shrimp*** | 0.0478 | 0.4435 | 84.3762 | 0.0448 | 0.33 | 80.9983 | **1.173** | **9.912** | 31.9167 |
| **Yellowfin Tuna** | 0.0477 | 0.4427 | 84.8189 | 0.0393 | 0.2891 | 81.2874 | 0.1122 | 0.9482 | 32.8649 |
| **Black Drum** | 0.0463 | 0.4300 | 85.2489 | 0.0911 | 0.6703 | 81.9577 | **0.1301** | **1.1** | 33.9649 |
| King Mackerel | 0.0453 | 0.4200 | 85.6689 | 0.0444 | 0.3271 | 82.2848 | 0.0114 | 0.0970 | 34.0619 |
| Greater Amberjack | 0.0420 | 0.3901 | 86.0590 | 0.0442 | 0.3255 | 82.6103 | 0.0099 | 0.0844 | 34.1463 |
| White Grunt | 0.0419 | 0.3883 | 86.4473 | 0.0258 | 0.1897 | 82.8 | <0.001 | 0.0040 | 34.1503 |
| Mojarras | 0.0382 | 0.3548 | 86.8021 | 0.0362 | 0.2665 | 83.0665 | <0.001 | <0.001 | 34.1503 |
| Caribbean Spiny Lobster | 0.0365 | 0.3390 | 87.1411 | 0.0281 | 0.2066 | 83.2731 | <0.001 | <0.001 | 34.1503 |
| Atlantic Thread Herring | 0.0347 | 0.3221 | 87.4632 | <0.001 | 0.0013 | 83.2744 | <0.001 | 0.0012 | 34.1515 |
| Mollusca | 0.0310 | 0.2878 | 87.7510 | 0.0995 | 0.7324 | 84.0068 | <0.001 | <0.001 | 34.1515 |
| Florida Stone Crab | 0.0188 | 0.1747 | 87.9257 | 0.0873 | 0.6427 | 84.6495 | 0.0016 | 0.0137 | 34.1652 |
| Sea Snails | 0.0135 | 0.1258 | 88.0515 | 0.0837 | 0.6158 | 85.2653 | <0.001 | <0.001 | 34.1652 |
| Mactra Surfclams | 0.0203 | 0.1890 | 88.2405 | 0.0706 | 0.5195 | 85.7848 | <0.001 | <0.001 | 34.1652 |
| Blue Runner | 0.0225 | 0.2085 | 88.4490 | 0.0491 | 0.3618 | 86.1466 | 0.0042 | 0.0362 | 34.2014 |
| Scombrids | 0.0100 | 0.0932 | 88.5422 | 0.0479 | 0.3529 | 86.4995 | <0.001 | 0.0018 | 34.2032 |
| ***Shrimps*** | 0.0345 | 0.3199 | 88.8621 | 0.0432 | 0.3181 | 86.8176 | 0.0513 | 0.4341 | 34.6373 |
| Cannonball Jellyfish | <0.001 | <0.001 | 88.8621 | 0.0431 | 0.3174 | 87.1350 | <0.001 | <0.001 | 34.6373 |
| Cobia | 0.0305 | 0.2832 | 89.1453 | 0.0377 | 0.2776 | 87.4126 | 0.0213 | 0.1806 | 34.8179 |
| Sand Weakfish | 0.0242 | 0.2248 | 89.3701 | 0.0366 | 0.2693 | 87.6819 | <0.001 | <0.001 | 34.8179 |
| ***Menhadens*** | 0.0047 | 0.0441 | 89.4142 | 0.0047 | 0.0441 | 87.7260 | **6.737** | **56.93** | 91.7479 |
| ***Crayfishes*** | <0.001 | <0.001 | 89.4142 | <0.001 | <0.001 | 87.7260 | <0.001 | <0.001 | 91.7479 |

| **Gulf of Mexico (cont’d)^[[11]](#footnote-11)^** | **Revenue** | | | | | | | | |
| --- | --- | --- | --- | --- | --- | --- | --- | --- | --- |
|  | **Pre-Katrina v. Post-Katrina/Pre-DWH** | | | **Pre-Katrina v. Post-DWH** | | | **Post-Katrina/Pre-DWH v. Post-DWH** | | |
| **Taxon** | **Av. dissim** | **Contrib. %** | **Cumul. %** | **Av. dissim** | **Contrib. %** | **Cumul. %** | **Av. dissim** | **Contrib. %** | **Cumul. %** |
| **Gulf Menhaden** | <0.001 | <0.001 | <0.001 | <0.001 | <0.001 | <0.001 | <0.001 | <0.001 | <0.001 |
| ***Eastern Oyster*** | **0.7827** | **4.959 (+)** | **4.959** | **2.789** | **12.31 (+)** | **12.31** | **2.35** | **13.66 (+)** | **13.66** |
| ***White Shrimp*** | **2.674** | **16.94 (+)** | 21.899 | **3.06** | **13.5 (+)** | 25.81 | **2.482** | **14.43 (+)** | 28.09 |
| **Atlantic Croaker** | 0.0202 | 0.1283 | 22.0273 | 0.0578 | 0.2552 | 26.0652 | 0.0423 | 0.2462 | 28.3362 |
| ***Misc. Crustaceans*** | 0.0178 | 0.113 | 22.1403 | 0.0158 | 0.0699 | 26.1351 | <0.001 | <0.001 | 28.3362 |
| **Spotted Seatrout** | 0.0257 | 0.1632 | 22.3035 | 0.0037 | 0.0167 | 26.1518 | 0.0052 | 0.0306 | 28.3668 |
| **Weakfishes** | <0.001 | <0.001 | 22.3035 | <0.001 | <0.001 | 26.1518 | <0.001 | <0.001 | 28.3668 |
| ***Blue Crab*** | **0.3957** | **2.507 (+)** | 24.8105 | **1.989** | **8.777 (+)** | 34.9288 | **1.803** | **10.48 (+)** | 38.8468 |
| **Striped Mullet** | **0.3392** | **2.149** | 26.9595 | **0.32** | **1.412** | 36.3408 | 0.0546 | 0.3176 | 39.1644 |
| **Porgies** | <0.001 | 0.0037 | 26.9632 | <0.001 | 0.0021 | 36.3429 | <0.001 | 0.0023 | 39.1667 |
| **Gag** | 0.0019 | 0.0124 | 26.9756 | 0.0043 | 0.0192 | 36.3621 | 0.0035 | 0.0206 | 39.1873 |
| **Atlantic Sea Bob** | **0.258** | **1.634** | 28.6096 | 0.2295 | 1.013 | 37.3751 | 0.0343 | 0.1999 | 39.3872 |
| ***Red Snapper*** | **0.2688** | **1.703** | 30.3126 | **0.5602** | **2.472 (+)** | 39.8471 | **0.7185** | **4.177 (+)** | 43.5642 |
| Dolphinfish | 0.0136 | 0.0865 | 30.3991 | 0.0121 | 0.0536 | 39.9007 | 0.0043 | 0.0254 | 43.5896 |
| **Sheepshead** | 0.0355 | 0.225 | 30.6241 | 0.0256 | 0.1132 | 40.0139 | 0.0168 | 0.0981 | 43.6877 |
| Calico Scallop | <0.001 | <0.001 | 30.6241 | <0.001 | <0.001 | 40.0139 | <0.001 | <0.001 | 43.6877 |
| **Red Drum** | 0.0012 | 0.0080 | 30.6321 | 0.0063 | 0.0282 | 40.0421 | 0.0067 | 0.0394 | 43.7271 |
| Red Grouper | <0.001 | <0.001 | 30.6321 | <0.001 | <0.001 | 40.0421 | <0.001 | <0.001 | 43.7272 |
| Spanish Mackerel | <0.001 | 0.0015 | 30.6336 | <0.001 | 0.0040 | 40.0461 | <0.001 | 0.0053 | 43.7325 |
| Mullets | 0.0043 | 0.0278 | 30.6614 | 0.0126 | 0.0558 | 40.1019 | 0.0097 | 0.0565 | 43.7890 |
| Northern Quahog | <0.001 | <0.001 | 30.6614 | <0.001 | <0.001 | 40.1019 | <0.001 | <0.001 | 43.7890 |
| Rock Shrimp | 0.0521 | 0.3302 | 30.9916 | 0.0498 | 0.2198 | 40.3217 | 0.0078 | 0.0455 | 43.8345 |
| Vermilion Snapper | 0.0468 | 0.2971 | 31.2887 | 0.0488 | 0.2157 | 40.5374 | 0.0704 | 0.4098 | 44.2443 |
| ***Brown Shrimp*** | **6.221** | **39.41** | 70.6987 | **5.418** | **23.91** | 64.4474 | **3.124** | **18.16 (+)** | 62.4043 |
| **Yellowfin Tuna** | **0.3575** | **2.265** | 72.9637 | **0.5687** | **2.51** | 66.9574 | 0.2906 | 1.69 | 64.0943 |
| **Black Drum** | <0.001 | <0.001 | 72.9637 | 0.1036 | 0.4571 | 67.4145 | <0.001 | <0.001 | 64.0943 |
| King Mackerel | 0.0575 | 0.3643 | 73.3280 | 0.1079 | 0.476 | 67.8905 | 0.0610 | 0.3547 | 64.4490 |
| Greater Amberjack | 0.0085 | 0.0541 | 73.3821 | 0.0083 | 0.0366 | 67.9271 | 0.0062 | 0.0360 | 64.4850 |
| White Grunt | <0.001 | <0.001 | 73.3821 | <0.001 | <0.001 | 67.9271 | <0.001 | <0.001 | 64.4850 |
| Mojarras | <0.001 | <0.001 | 73.3821 | <0.001 | <0.001 | 67.9271 | <0.001 | <0.001 | 64.4850 |
| Caribbean Spiny Lobster | <0.001 | <0.001 | 73.3821 | <0.001 | <0.001 | 67.9271 | <0.001 | <0.001 | 64.4850 |
| Atlantic Thread Herring | <0.001 | <0.001 | 73.3821 | <0.001 | <0.001 | 67.9271 | <0.001 | <0.001 | 64.4850 |
| Mollusca | <0.001 | <0.001 | 73.3821 | <0.001 | <0.001 | 67.9271 | <0.001 | <0.001 | 64.4850 |
| Florida Stone Crab | <0.001 | <0.001 | 73.3821 | 0.0149 | 0.0659 | 67.9930 | 0.0058 | 0.0342 | 64.5192 |
| Sea Snails | <0.001 | <0.001 | 73.3821 | <0.001 | <0.001 | 67.9930 | <0.001 | <0.001 | 64.5192 |
| Mactra Surfclams | <0.001 | <0.001 | 73.3821 | <0.001 | <0.001 | 67.9930 | <0.001 | <0.001 | 64.5192 |
| Blue Runner | 0.0028 | 0.0182 | 73.4003 | 0.0034 | 0.0152 | 68.0082 | 0.0013 | 0.0080 | 64.5272 |
| Scombrids | 0.0010 | 0.0069 | 73.4072 | <0.001 | 0.0042 | 68.0124 | <0.001 | <0.001 | 64.5272 |
| ***Shrimps*** | 0.0535 | 0.3392 | 73.7464 | **0.3648** | **1.61 (+)** | 69.6224 | **0.3867** | **2.248 (+)** | 66.7752 |
| Cannonball Jellyfish | <0.001 | <0.001 | 73.7464 | <0.001 | <0.001 | 69.6224 | <0.001 | <0.001 | 66.7752 |
| Cobia | 0.0046 | 0.0293 | 73.7757 | 0.0032 | 0.0145 | 69.6369 | 0.0026 | 0.0156 | 66.7908 |
| Sand Weakfish | <0.001 | <0.001 | 73.7757 | <0.001 | <0.001 | 69.6369 | <0.001 | <0.001 | 66.7908 |
| ***Menhadens*** | **1.442** | **9.135** | 82.9107 | **3.85** | **16.99 (+)** | 86.6269 | **4.191** | **24.36 (+)** | 91.1508 |
| ***Crayfishes*** | **1.582** | **10.022 (+)** | 92.9327 | **1.6385** | **7.23 (+)** | 93.8569 | **0.4652** | **2.705 (+)** | 93.8558 |

Average dissimilarity (Av. Dissim), percent contribution (Contrib. %), and cumulative percent contribution (Cumul. %) for all major species (i.e.,

cumulatively up to 90% through SIMPER analysis) contributing to significant differences in species composition for biomass, landings, and revenue among

pre-event, event, and post-event periods (as examined through analysis of similarity testing). For eastern Bering Sea (EBS), Gulf of Alaska (GAK), northern

California, and Pacific Northwest ecosystems, tests of significance in values were conducted for time periods ten years prior to the Pacific marine heatwave

(“Blob”), over the duration of the heatwave, and post-heatwave. For the Gulf of Maine, differences in values were examined among time periods ten years

prior to the onset of an accelerated warming period for the Gulf of Maine, during the accelerated warming period and prior to a subsequent marine heatwave

and noted spike in temperatures, and for years following the heatwave and during the temperature spike. For the northern Gulf of Mexico, tests were

conducted for time periods ten years prior to Hurricane Katrina, during the post-hurricane period prior to the Deepwater Horizon (DWH) oil spill, and post-

DWH event. All species with Contrib. % values greater than 1.0 are in bold. Italicized species names indicate that a mean increase (shown as +) was observed

between at least one period for a species with a Contib. % greater than 1.0 for a given variable.

1. Overall Av. Dissim. - Revenue (Pre-Heatwave v. Post-Heatwave): 23.4 [↑](#footnote-ref-1)
2. Overall Av. Dissim. - Landings (Pre-Heatwave v. Post-Heatwave): 19.5; Overall Av. Dissim. - Revenue (Pre-Heatwave v. Post-Heatwave): 32.2 [↑](#footnote-ref-2)
3. Overall Av. Dissim. - Biomass (Pre-Heatwave v. Heatwave): 37.4; Biomass (Pre-Heatwave v. Post-Heatwave): 50.3; Landings (Pre-Heatwave v. Post-Heatwave): 50.7 [↑](#footnote-ref-3)
4. Overall Av. Dissim. - Revenue (Pre-Heatwave v. Heatwave): 32.1 [↑](#footnote-ref-4)
5. Overall Av. Dissim. - Biomass (Pre-Heatwave v. Post-Heatwave): 28.5; Landings (Pre-Heatwave v. Post-Heatwave): 34.3; Landings (Heatwave v. Post-Heatwave): 27.6 [↑](#footnote-ref-5)
6. Overall Av. Dissim. - Revenue (Pre-Heatwave v. Heatwave): 24.3 [↑](#footnote-ref-6)
7. Overall Av. Dissim. - Biomass (Pre-Accel v. Post-Accel/Post-Heatwave): 33.2 [↑](#footnote-ref-7)
8. Overall Av. Dissim. - Landings (Pre-Accel v. Post-Accel/Post-Heatwave): 39.4; Landings (Pre-Accel v. Accel/Pre-Heatwave): 25.5; Landings (Accel/Pre-Heatwave v. Post-Accel/Post-Heatwave): 26.7 [↑](#footnote-ref-8)
9. Overall Av. Dissim. - Revenue (Pre-Accel v. Post-Accel/Post-Heatwave): 37.7; Revenue (Accel/Pre-Heatwave v. Post-Accel/Heatwave): 21.9 [↑](#footnote-ref-9)
10. Overall Av. Dissim. - Biomass (Pre-Katrina v. Post-Katrina/Pre-DWH): 10.8; Biomass (Pre-Katrina v. Post-DWH): 13.6; Landings (Pre-Katrina v. Post-DWH): 11.8 [↑](#footnote-ref-10)
11. Overall Av. Dissim. - Revenue (Pre-Katrina v. Post-Katrina/Pre-DWH): 15.8; Revenue (Pre-Katrina v. Post-DWH): 22.7; Revenue (Post-Katrina/Pre-DWH v. Post-DWH): 17.2 [↑](#footnote-ref-11)
